# Development of a quantitative self-assessment tool for hospital antimicrobial stewardship and infection control programs: a step towards standardizing clinical studies

**DOI:** 10.1093/jacamr/dlag013

**Published:** 2026-02-06

**Authors:** V Zanichelli, S Z Zakariah, A Y Classen, U Dumpis, C G Giske, S Goepel, D Hagen, S B Jorgensen, J Kessel, C Kjellander, L K S Kleppe, G S Simonsen, M J G T Vehreschild, J J Vehreschild, M Semret, for the PILGRIM Study Group, Pauls Aldins, Per Espen Akselsen, Anne Mette Asfeldt, Lena Biehl, Nadine Conzelmann, Kelly Davison, Thilo Dietz, Simone Eisenbeis, Lucas J Fein, Fe Dja Farowski, Romina Georghe, Maayan Huberman Samuel, Barbara Ann Jardin, Merve Kaya, Leonard Leibovici, Zane Linde Ozola, Noa Eliakim Raz, Nick Schulze, Hannes Wåhlin, Aija Vilde, Viesturs Zvirbulis

**Affiliations:** McGill University Health Centre, Department of Medicine, Infectious Diseases, Montreal, Quebec, Canada; McGill University Health Centre, Department of Medicine, Infectious Diseases, Montreal, Quebec, Canada; Universiti Putra Malaysia, Medical Microbiology Department, Serdang, Selangor, Malaysia; Department I of Internal Medicine, University of Cologne, Faculty of Medicine and University Hospital of Cologne, Cologne, Germany; German Centre for Infection Research, Partner Site Bonn-Cologne, Cologne, Germany; Pauls Stradins Clinical University Hospital, University of Latvia, Riga, Latvia; Division of Clinical Microbiology, Department of Laboratory Medicine, Karolinska Institutet and Department of Clinical Microbiology, Karolinska University Hospital, Stockholm, Sweden; Infectious Diseases, Department of Internal Medicine I, University Hospital Tübingen, Tübingen, Germany; Haukeland University Hospital, University of Bergen, Bergen, Norway; Department of Emergency Medicine and Department of Microbiology and Infection Control, Akershus University Hospital, Lorenskog, Norway; Goethe University, Department of Internal Medicine 2, Infectious Diseases, Frankfurt, Germany; Department of Laboratory Medicine, Karolinska Institutet and Department of Internal Medicine, Capio St Göran Hospital, Stockholm, Sweden; Department of Infectious Diseases and Department for Infection Control and Prevention, Stavanger University Hospital, Stavanger, Norway; Department of Microbiology and Infection Control, University Hospital of North Norway and Faculty of Health Sciences, UiT Arctic University of Norway, Tromsø, Norway; Department II of Internal Medicine, Infectious Diseases, Goethe University Frankfurt, University Hospital Frankfurt, Frankfurt am Main, Germany; Department I of Internal Medicine, University of Cologne, Faculty of Medicine and University Hospital of Cologne, Cologne, Germany; Goethe-Universität Frankfurt, Fachbereich Medizin, Institut für Digitale Medizin und Klinische Datenwissenschaften, Frankfurt, Germany; McGill University Health Centre, Department of Medicine, Infectious Diseases, Montreal, Quebec, Canada

## Abstract

**Background:**

Antimicrobial stewardship (AMS) and infection prevention and control (IPC) programs are crucial for reducing antimicrobial resistance in hospitals. Existing quality indicators (QIs) for these programs are mainly qualitative, hindering external benchmarking. PILGRIM (NCT03765528) is a prospective multinational cohort study evaluating the impact of antibiotic prescription quality on intestinal domination by healthcare-associated pathogens.

**Objective:**

In this sub-study, we develop a quantitative scoring tool for AMS and IPC programs to facilitate standardized assessments of programs and support clinical studies.

**Methods:**

We used a RAND-modified Delphi consensus procedure to establish a scoring system for AMS and IPC programs. The tool was tested using data collected from eight hospitals in five countries during 2019–2024. We evaluated temporal associations between scores, *Clostridioides difficile* cases, hand disinfectant and antibiotic use.

**Results:**

We assessed 98 QIs, resulting in in a final set of 62 QIs (35 for AMS and 27 for IPC). For our sites, the overall median score was 29 out of 50 (IQR 28–31) for AMS and 36 out of 50 (IQR 33–38) for IPC programs. Higher-scoring sites decrease antibiotic use over time. IPC scores were positively correlated with hand disinfectant use.

**Conclusion:**

This quantitative scoring scheme represents a promising step towards standardizing assessments of AMS and IPC programs in high-income settings, enabling external comparisons and supporting future clinical studies. Further validation is needed to refine its predictive validity and ensure its utility in diverse healthcare settings.

## Introduction

Antimicrobial stewardship (AMS) programs in acute-care hospitals (hospitals that provide active short-term treatment for injuries, a disease or severe episode of illness) are essential for promoting appropriate antimicrobial use, maximizing treatment efficacy and minimizing selection of antimicrobial-resistant (AMR) microorganisms.^1^

When combined with effective infection prevention and control (IPC) programs, their impact on reducing the prevalence of AMR microorganisms and antimicrobial use has been well documented.^2,3^ Recognizing the need to tailor programs to available resources, frameworks combining quality indicator (QI) sets in hospital settings provide structured mechanisms to evaluate capacity—though their applicability varies considerably, as highlighted in a recent systematic review of 229 QIs across 16 studies.^4^ Notably, most existing frameworks lack a numerical scoring component to reflect the degree of achievement of the indicators; although they can support internal monitoring over time, they are generally not designed for external comparisons or benchmarking across institutions.^5–9^ Furthermore, integrated IPC and AMS frameworks are lacking despite the complementarity and even synergy of these programs, limiting their utility in assessing real-world performance of programs on drug-resistant infections.

With growing interest in multicentre clinical studies focused on AMR reduction, there is a need for robust, interpretable tools that can support the validation and clinimetric evaluation of QIs and facilitate intersetting comparisons. PILGRIM (registered under ClinicalTrials.gov; NCT03765528) is a prospective observational cohort study conducted in 10 hospital sites across 6 high-income countries, with the aim of evaluating the impact of antibiotic prescription quality on intestinal colonization and domination by healthcare-associated pathogens.^10,11^ In this sub-study, we develop and test a numerical scoring tool for AMS and IPC programs that is acceptable to end users, sensitive to temporal changes, suitable for validation as a credible measure of hospital program quality or performance and, therefore, can serve as a tool to assess ‘site-readiness’ for AMR-related clinical research. Using QIs extracted from published implementation checklists, we employed a consensus-based approach to develop AMS and IPC scoring systems based on the predicted impact on clinically meaningful outcomes. We then tested the scores obtained for each participating hospital against antibiotic consumption and *Clostridioides difficile* cases over time.

## Methods

### Description of the study sites

The study was conducted across eight acute-care hospital sites that varied in size, infrastructure age and clinical focus. The participating hospitals ranged from 499 to 1589 beds and included both medical and surgical services. Wards represented included haematology-oncology, stem cell transplantation, cardiology, cardiothoracic surgery, urology, gastrointestinal surgery, rheumatology and infectious diseases.

The age of the main hospital buildings ranged from 3 to 65 years, reflecting heterogeneity in infrastructure. Average length of stay across sites varied between 3.6 and 6.7 days, where available. Each site contributed between two and three wards to the study, which were assigned site-specific ward identifiers (ID1–ID3) for analytic purposes. A summary of site characteristics is provided in Table 1.

**Table 1.** Characteristics of the hospitals that participated in the self-assessment exercise

Site	Wards included	Hospital size (number of beds)	Age of the main hospital building (years)	Average length of stay (days)
Site 1	Haematology-Oncology (ID1), Cardiology (ID2)	517	3	6.7
Site 2	Oncology (ID1), Haematology (ID2), Cardiothoracic Surgery (ID3)	499	29	4.6
Site 3	Urology (ID1), Gastrointestinal Surgery (ID2), Haematology (ID3)	705	11	3.6
Site 4	Surgery (ID1), Haematology-Oncology (ID2)	1589	65	6.4
Site 5	Urology (ID1), Cardiac Surgery (ID2)	981	43	3.9
Site 6	Haematology-Rheumatology (ID1), Stem Cell Transplant (ID2), Infectious Disease (ID3)	1488	49	6.4
Site 7	Oncology (ID1), Cardiothoracic Surgery (ID2)	900	36	4
Site 8	Gastrointestinal Surgery (ID1), Haematology (ID2)	587	37	Not available

ID, indicator department.

### RAND-modified Delphi procedure to select quality indicators

We used a modified Delphi approach to develop a list of QIs for AMS and IPC programs and a standardized numerical scoring scheme for each QI in an empirical but systematic fashion. The procedure consisted of a four-round online survey or face-to-face meetings to establish a consensus. All face-to-face meetings were conducted online via video conference.

Investigators of the PILGRIM consortium with expertise in infectious diseases and/or AMS, infection control or clinical microbiology were invited by email to participate. Experts were asked to evaluate the importance of a list of indicators prepared by two of the study investigators (M.S. and S.Z.Z.) based on a literature review of existing tools and their personal experience in the field.^12–15^

The QIs were presented to experts to be grouped into domains. Domains were defined as broad categories of actions or strategies within a program, whereas QIs were described as specific actions/interventions within a domain. In the first round, experts were asked to rate the appropriateness of their inclusion and the level of importance of each domain in terms of potential impact on clinically meaningful outcomes and of each QI. Indicators were rated using a 5-point Likert scale ranging from 1 ‘completely disagree’ to 5 ‘completely agree’ and domains were assigned weights (e.g. <25%, 25%–50% or 50%–70% of the total) to reflect their relative importance within the assessment tool. During the second round, discordances on QIs and domains were discussed. Discordance was defined as <100% agreement within the group (e.g. at least one expert choosing ‘somewhat disagree’ or ‘completely disagree’). Discordances were discussed until complete agreement was reached (i.e. accept/reject or modify). In the third round, a numerical scoring scheme for each accepted QI was proposed ranging from 0 ‘not in place-absent’, to 2 ‘fully implemented-present’. For example, if a hospital has an established AMS leadership team, this indicator would be scored as ‘present,’ whereas if no such structure exists, it would be ‘absent’.

Experts were asked to agree or disagree with the proposed scoring scheme and to comment on the definitions assigned to ‘yes’, ‘partial’ and ‘no’ answers. A fourth round of talks was held to discuss significant discordances until complete agreement was reached.

### Testing

During the period of active patient recruitment for the main PILGRIM study (2019–2022), each study site had completed a survey questionnaire describing existing IPC and AMS programs in place, data on hand disinfectant consumption when available [volume (mL used per patient-day for each indicator department), number of *C. difficile* infection (CDI) cases (as defined by local testing practices) and hospital antibiotic use in DDD per 100 bed-days]. Data on duration of programs, whether they underwent formal quality audits and the specific type of disinfectants (brand) were not available. The self-assessment tool consisted of a blank template containing all the QI, scoring rules and domains retained by consensus. A team member blinded to the sites then converted survey responses to tabulated domain-specific and overall numerical scores for IPC and AMS programs for each site and for each year of the study.

Team leaders for each study site were asked to review their tabulated scores for the years 2019–2022 and then to use the tool to score their sites for the year 2024. When study team leaders were not responsible for IPC or AMS at their sites, they were asked to review their scores with the responsible leads. Any score differences in 2024 compared to previous years were discussed by email to determine whether these reflected a true change in program activities or were due to interpretation errors.

### Association between scores and outcomes of interest

We explored potential temporal associations between AMS and IPC program scores, total antibiotic use, hand disinfectant consumption and CDI using simple scatter plots to visually represent relationships between these variables over time. We postulated that total antibiotic use would remain stable or decrease over time in sites with high AMS scores and increase over time in sites with low scores. Given the small number of data points, no statistical analyses or correlation coefficients were conducted.

### Ethics and regulatory status

The study was reviewed and approved by the responsible ethics committees and/or institutional review boards of all participating sites.

## Results

### RAND-modified Delphi procedure

Of the nine PILGRIM principal investigators (i.e. study site leaders) invited to participate, eight accepted the invitation and were included in the panel. Two of the study investigators (S.Z.Z. and M.S.) moderated the panel and meetings took place between January and March 2024. All panel members had clinical experience as IPC or AMS experts working in high-income settings (not necessarily hospital leads for these activities); two of the experts (S.Z.Z. and M.S.) had additional experiences in low- and middle-income countries.

In Round 1, 54 of the 98 QIs (20/60 IPC; 34/38 AMS) reached complete agreement and were accepted with no further discussion. The remaining 44 QIs (44.9%) moved to Round 2 (40 IPC; 4 AMS). Disagreements reflected difficulty in distinguishing levels of agreement (e.g. ‘generally’ versus ‘completely agree’), wording issues (e.g. use of ‘very important’) or the need to consider the local epidemiology (e.g. screening for multidrug-resistant organisms dependent on prevalence). In Round 2, for IPC, 2 indicators were accepted unchanged, 8 were combined into four, 1 was rephrased and 29/40 were rejected (Table S1, available as Supplementary data at *JAC-AMR* Online). For AMS, one indicator was eliminated and three related to antibiotic consumption were combined into one (Table 2). At the end of Round 2, consensus was reached on 62 QIs (27 IPC; 35 AMS) organized in 10 domains (4 IPC; 6 AMS). In Round 3, scoring was defined for each indicator as 2 for fully achieved, 1 for partially achieved and 0 for not achieved yielding a total possible score of 0–50 for both IPC and AMS. The final list of quality indicators and scores are presented in Table 2 (IPC) and Table 3 (AMS).

**Table 2.** List of IPC quality indicators and assigned scores

Quality indicator (QI)	Domain	Score (with instructions were applicable)			QI
1. Are all patient rooms single occupancy?	Structure	2 > 50% rooms	1 10–49%	0 < 10%	IPC QI1
2. Is every patient room equipped with one bathroom \including sink, toilet and shower?	Structure	2 all rooms	1 some rooms/all rooms partially equipped	0	IPC QI2
3. Are disinfectant dispensers available in each patient room?	Structure	2 all rooms	1 some rooms	0	IPC QI3
4. Full time equivalents (FTEs): nurses (nurse:patient ratio)	Structure	2 ≥ 1:5	1 1:6	0 <1:6	IPC QI4
Pathogen: VRE (QI 5–7)					
5. Do you regularly^a^ screen patients for VRE carriage?	Precautions	2 all	1 only those with certain criteria	0	IPC QI5
6. Single room contact precautions applied?	Precautions	2 all	1 cohorting	0	IPC QI6
7. Do you wear gloves and gowns before direct patient contact?	Precautions		1 gloves and gowns	0 gowns only or none	IPC QI7
Pathogen: EPE (QI 8–10)					
8. Do you regularly^a^ screen patients for EPE carriage?	Precautions	2 all	1 only those with certain criteria	0	IPC QI8
9. Single room contact precautions applied?	Precautions	2 all	1 cohorting	0	IPC QI9
10. Do you wear gloves and gowns before direct patient contact?	Precautions		1 gloves and gowns	0 gown only or none	IPC QI10
Pathogen: CRE/CPE (QI 11–13)					
11. Do you regularly^a^ screen patients for CRE/CPE carriage?	Precautions	2 all	1 only those with certain criteria	0	IPC QI11
12. Single room contact precautions applied?	Precautions	2	1	0	IPC QI12
13. Do you wear gloves and gowns before direct patient contact?	Precautions		1 gloves and gowns	0 gown only or none	IPC QI13
Pathogen: *C. difficile* (QI 14 and 15)					
14. Single room contact precautions applied?	Precautions	2 all	1 cohorting	0	IPC QI14
15. Do you wear gloves and gowns before direct patient contact?	Precautions		1 gloves and gowns	0 gown only or none	IPC QI15
16. Frequency of cleaning of high touch objects in patient room	Cleaning	2 twice a day	1 once daily	0 if necessary/on demand	IPC QI16
17. Frequency of cleaning of other objects/spots in patient room	Cleaning	2 twice a day	1 once daily	0 if necessary/on demand	IPC QI17
18. Cleaning of spots close to patient bed performed by nurses or cleaning staff	Cleaning	2 by cleaning staff	1 by nurses	0	IPC QI18
19. Are there cleaning standards available for the cleaning of rooms after patient discharge, if the patients were not colonized or infected with a multiresistant pathogen	Cleaning	2 proper disinfectant	1 without proper disinfectant	0	IPC QI19
20. Are there cleaning standards available for the cleaning of rooms after patient discharge, if the patients were colonized or infected with a multiresistant pathogen/*C. difficile*?	Cleaning	2 proper disinfectant	1 without proper disinfectant	0	IPC QI20
21. Are there routinely performed cleaning controls by the hygiene department or the cleaning company?	Cleaning	2		0	IPC QI21
22. Are there training courses for the cleaning staff provided by the hygiene department or the cleaning company?	Cleaning	2		0	IPC QI22
23. Is hand disinfectant consumption (mL/patient-day) assessed for this department?	Cleaning	2		0	IPC QI23
24. Is there clear visible information on hand hygiene procedures (e.g. poster on ‘5 moments for hand hygiene’ of the WHO) in at least three important/visible points at the hospital ward?	Hand hygiene	2		0	IPC QI24
25. Are there regularly provided trainings on hand hygiene procedures for healthcare professionals?	Hand hygiene	2		0	IPC QI25
26. Are there regularly performed (at least once per year) audits of hand hygiene compliance?	Hand hygiene	2		0	IPC QI26
27. Is feedback provided to hospital staff after evaluation of hand hygiene compliance?	Hand hygiene	2		0	IPC QI27

CPE, carbapenemase-producing Enterobacterales; CRE, carbapenem-resistant Enterobacterales; EPE, extended-spectrum β-lactamase producing Enterobacterales; VRE, vancomycin-resistant Enterococcus.

^a^‘Regularly’ refers to a recurrent, structured activity irrespective of the exact frequency.

**Table 3.** List of AMS quality indicators and assigned scores

Quality indicators (QIs)	Domain	Score	QI		
1. Does your facility have a formal, written statement of support from leadership that supports efforts to improve antibiotic use?	Structure	1		0	AMS QI01
2. Does your facility receive any budgeted financial support for antibiotic stewardship activities (e.g. support for salary, training or IT support)?	Structure	1		0	AMS QI02
3. Is there a physician leader responsible for program outcomes of stewardship activities at your facility?	Structure	1		0	AMS QI03
4. Is there a pharmacist leader responsible for working to improve antibiotic use at your facility?	Structure	1		0	AMS QI04
Does any of the staff below work with the stewardship leaders to improve antibiotic use?					
5. Clinicians	Structure	1		0	AMS QI05
6. Microbiology (laboratory)	Structure	1		0	AMS QI06
7. Any of IPC, information technology (IT), nursing, QI, others	Structure	1		0	AMS QI07
8. Does your facility have a policy that requires prescribers to document in the medical record or during order entry a dose, duration and indication for all antibiotic prescriptions?	Guideline/policy	2 dose, duration and indication for all prescription	1 dose and duration only or some prescriptions	0	AMS QI08
9. Does your facility have facility-specific treatment recommendations, based on national guidelines and local susceptibility, to assist with antibiotic selection for common clinical conditions?	Guideline/policy	2 facility-specific recommendations for most common indications	1 facility-specific recommendations for some or only national recommendations	0	AMS QI09
10. Is there a formal procedure for all clinicians to review the appropriateness of all antibiotics 48 h after the initial orders (e.g. antibiotic time out)?	Guideline/policy	2 review all antibiotics after 48–72 h	1 review of only some antibiotics or after 3–7 days	0	AMS QI10
11. Do specified antibiotic agents need to be approved by a physician or pharmacist prior to dispensing (i.e. pre-authorization) at your facility?	Guideline/policy	1		0	AMS QI11
12. Does a physician or pharmacist review courses of therapy for specified antibiotic agents (i.e. prospective audit with feedback) at your facility?	Interventions	3 Prospective reviews weekly	2 Prospective but >weekly	0	AMS QI12
			1 Retrospective audits		
13. Automatic changes from intravenous to oral antibiotic therapy in appropriate situations?	Interventions	1		0	AMS QI13
14. Dose adjustments in cases of organ dysfunction?	Interventions	1		0	AMS QI14
15. Dose optimization (pharmacokinetics/pharmacodynamics) to optimize the treatment of organisms with reduced susceptibility?	Interventions	1		0	AMS QI15
16. Automatic alerts in situations where therapy might be unnecessarily duplicative?	Interventions	1		0	AMS QI16
Specific interventions to ensure optimal treatment of					
17. Community-acquired pneumonia (CAP)	Interventions	1		0	AMS QI17
18. Urinary tract infections (UTI)	Interventions	1		0	AMS QI18
19. Skin and soft tissue infections	Interventions	1		0	AMS QI19
20. Surgical prophylaxis	Interventions	1		0	AMS QI20
21. Empirical treatment of methicillin-resistant *Staphylococcus aureus* (MRSA)	Interventions	1		0	AMS QI21
22. Non-CDI antibiotics in new cases of CDI (e.g. review of all CDI to stop unnecessary antibiotics)	Interventions	1		0	AMS QI22
23. Culture-proven invasive (e.g. bloodstream) infections	Interventions	1		0	AMS QI23
24. Does your stewardship program monitor adherence to a documentation policy (dose, duration and indication)?	Monitoring	2 adherence good/very good	1 adherence insufficient	0	AMS QI24
25. Does your stewardship program monitor adherence to facility-specific treatment recommendations?	Monitoring	2 adherence good/very good	1 adherence insufficient	0	AMS QI25
26. Does your stewardship program monitor compliance with one or more of the specific interventions in place?	Monitoring	2 adherence good/very good	1 adherence insufficient	0	AMS QI26
27. Does your facility track rates of *C. difficile* infection?	Surveillance	2		0	AMS QI27
28. Does your facility monitor antibiotic use (consumption) by counts of antibiotic(s) administered to patients per day (days of therapy, DOT) and/or by DDD and/or by expenditure?	Surveillance	2 by DOT or DDD	1 by expenditure only	0	AMS QI28
29. Does your facility monitor antibiotic use (consumption) at the unit level	Surveillance	2 annually	1 <annually	0	AMS QI29
30. Does your facility monitor antibiotic use (consumption) at the facility level	Surveillance	2 annually	1 <annually	0	AMS QI30
31. Does your facility produce an antibiogram (cumulative antibiotic susceptibility report)?	Reporting/education/training	2 annually	1 <annually	0	AMS QI31
32. Does your stewardship program share facility-specific reports on antibiotic use with prescribers?	Reporting/education/training	2 annually	1 <annually	0	AMS QI32
33. Has a current antibiogram been distributed to prescribers at your facility?	Reporting/education/training	1		0	AMS QI33
34. Do prescribers ever receive direct, personalized communication about how they can improve their antibiotic prescribing?	Reporting/education/training	2 immediate feedback	1 aggregate or for some indications/antibiotics	0	AMS QI34
35. Does your stewardship program provide education to clinicians and other relevant staff on improving antibiotic prescribing?	Reporting/education/training	1		0	AMS QI35

### Testing of scoring tools

IPC and AMS programs of each participating PILGRIM sites (described in Table 1) were scored using the QI scoring scheme developed during the consensus procedure, for the period 2019–2024.

In total, 8 hospitals in 5 countries reported data for a total of 22 departments. Seven of these eight sites reported data for at least 3 years, while one site reported data for only 2 years of the study.

### IPC scores

Across all eight participating sites, the overall median score for IPC was 36 [interquartile range (IQR) 33–38, maximum score 50]. There was some variation between departments within some hospital sites in terms of structure (number of single rooms, presence of dedicated bathroom, nurse:patient ratio and hand hygiene), but overall little variation between departments in terms of ‘Precautions’ (indicators for screening and contact precautions) and cleaning (Table S3).

Overall and domain-specific scores for all sites (median and IQR) are presented in Table 4. Details of the scores for each site are presented in Table S3.

**Table 4.** IPC and AMS programs self-assessment scores (all sites)

	Domain	Maximum possible score	Median score (IQR) (all sites)
IPC	Structure	8	7 (IQR 6–7)
	Precautions	18	13.5 (IQR 10–14)
	Cleaning	16	13 (IQR 11–13)
	Hand hygiene	8	5 (IQR 3–6)
	Total IPC score	50	36 (IQR 33–38)
AMS	Structure	7	7 (IQR 5–7)
	Guidelines	7	5 (IQR 3–5)
	Interventions	14	3 (IQR 1–4)
	Monitoring and surveillance	14	9 (IQR 8–10)
	Reporting and education	8	6 (IQR 5–7)
	Total AMS score	50	29 (IQR 28–31)

AMS, antimicrobial stewardship; IPC, infection prevention and control; IQR, interquartile range.

The median score was calculated using the data for each department for the years 2019 to 2024 (i.e. multiple years and multiple departments).

Among the seven sites with ≥3 years of data, four showed stable IPC scores over time (≤2-point change), while three showed notable variation: Sites 3 and 7 improved driven by higher hand hygiene scores, whereas Site 5 declined due to worsening indicators across all domains.

### AMS scores

Across all eight participating hospital sites, the overall median score for AMS was 29 (IQR 28–31). Total scores ranged from 23 to 40 (all years combined, maximum score 50). Overall and domain-specific scores for all sites (median and IQR) are presented in Table 4. Details of the scores at each site are presented in Table S4.

All sites performed well in the ‘Structure’ domain, with four (Sites 3, 6, 7 and 8) consistently achieving the maximum score. Except for Site 3, all sites showed changes between 2019 and 2024; Site 6 improved, while Site 5 declined. The most variability between sites was in the domain ‘Interventions’ (range 1–9, maximum 14) and ‘Reporting and education’ (median score 6, range 4–8, maximum 8).

The sites with the highest score for ‘Interventions’ reported specific strategies for surgical prophylaxis, bloodstream infections, dose adjustments in case of organ dysfunction, dose optimization particularly for resistant pathogens and regularly performed prospective audits with feedback. The site with the lowest score reported conducting only sporadic audit and feedback activities and no other interventions. Prospective audit and feedback activities were reported by six sites in 2024, but only two performed these consistently on a weekly basis (Sites 1 and 8).

For the domain ‘Guidelines and Policy’, the median score was 5 (range 2–5, maximum 7) and was mostly stable over time with three of the four sites with the highest score reporting no change between 2019 and 2024. One of the sites with the highest score in this domain reported an increase in 2024 (from 3 to 5) as they started a policy that required prescribers to document details of the antibiotic prescription in the medical record and a formal procedure to review prescriptions after 48 h. For ‘Monitoring and surveillance’, the median score was high (9, range 8–14, maximum 14); most sites noted some improvement in 2024.

**Figure 1.**
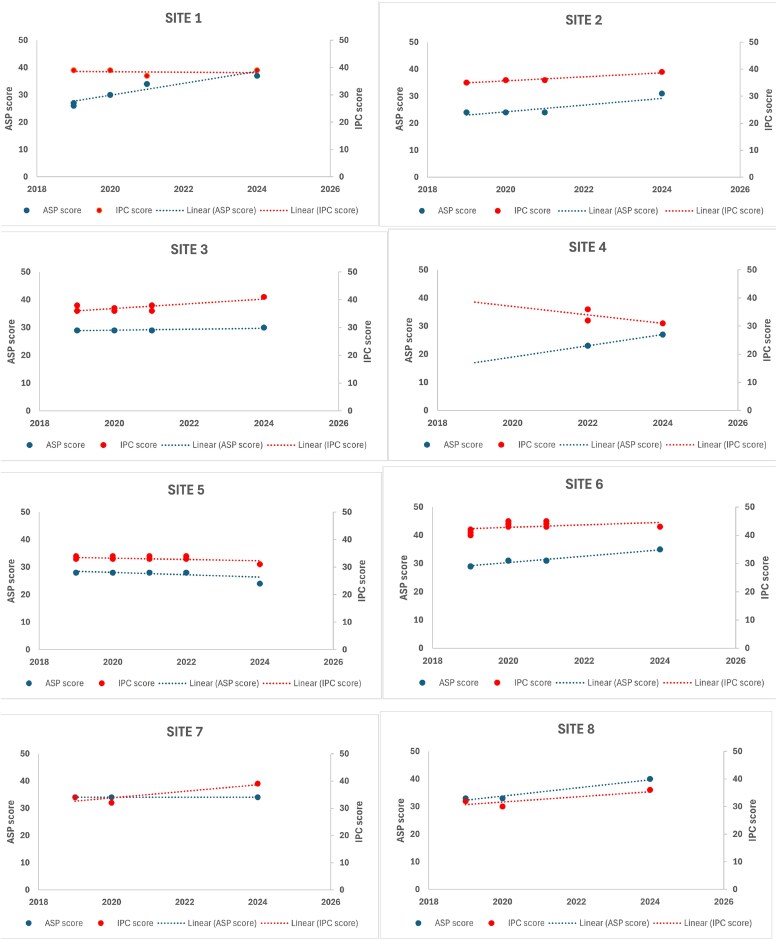
Temporal patterns in AMS and IPC program scores across study sites.

Figure 1 presents scores at various time points for each participating site.

### Total antibiotic consumption and AMS scores

**Figure 2.**
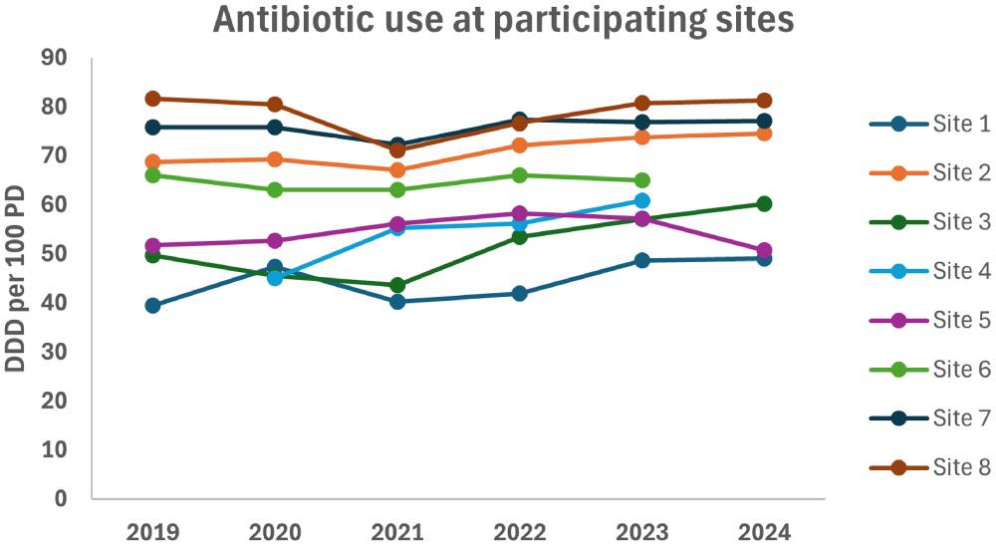
Total antimicrobial use at each participating site over time (expressed in DDD per 100 patient-days).

**Figure 3.**
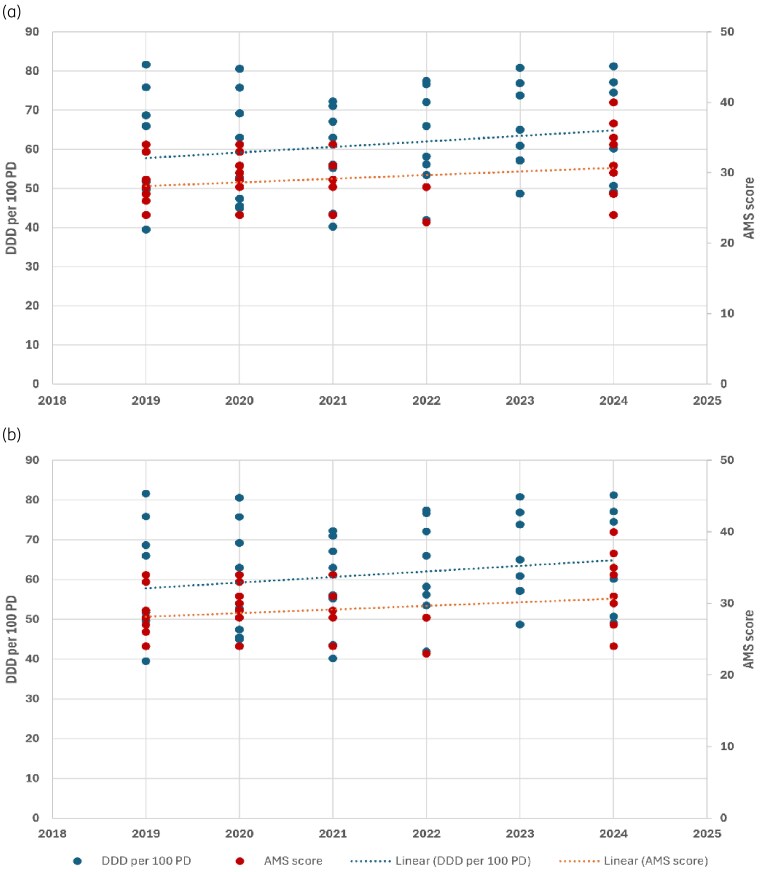
Temporal association between annual antibiotic use and site assessment scores. (a) Sites scoring in the upper quartile for AMS (AMS scores ≥31). (b) All other sites (AMS scores ≤30).

Total antibiotic use data were available for all participating hospitals from 2019 to 2024 (Figure 2), with two sites (Sites 4 and 6) not reporting data for 2024. In five of the eight sites, antibiotic use increased over the study period, though there was a decline in 2021 (peak of the COVID-19 pandemic) in some sites. Mean antibiotic use increased from 62 DDDs per 100 patient-days in 2019 (range across sites: 39–81) to 65 DDDs per 100 patient-days in 2024 (range: 49–81). Figure 3 represents visual trends in total antibiotic use and AMS scores from 2019 to 2024. For sites in the top quartile for stewardship (scores ≥31), there was a visual trend towards further strengthening of programs and decrease in antibiotic use. For sites with scores <30, there was little improvement in scores over time and an upward visual trend in antibiotic use. Site-specific associations between total antibiotic use and stewardship scores (including with the domain ‘Interventions’) are shown in Figures S2 and S3.

### New CDIs and total scores

New CDI data were only available as the absolute (total recorded) number of cases per year at the hospital level and not rates, and only three sites (Sites 1, 2 and 3) sent data for more than 2 years. No clear temporal pattern was detected between the number of new CDIs and the total AMS or IPC scores. These data are reported in Figure S4.

### Hand disinfectant consumption, hand hygiene and IPC scores

Consumption of hand disinfectant (mL/patient-day) at the indicator department level was available for at least 2 years for most sites (7/8). Total IPC scores, which were closely aligned with hand hygiene scores, tended to visually correlate with disinfectant use in all sites (shown in Figures S5 and S6).

## Discussion

In this study, we developed a quantitative scoring system to assess hospital AMS and IPC programs and standardize site selections for AMR-related clinical studies. This approach may help reduce inefficiencies in trial conduct by ensuring that participating sites have the necessary pragmatic capacities.

We incorporated interventions previously shown to improve antibiotic prescribing practices into the AMS scoring scheme^16^ and aligned the IPC scoring scheme with multimodal strategies included in the WHO Infection Prevention and Control Assessment Framework (IPCAF).^5^ In previous studies, high IPCAF scores have been associated with lower rates of hospital-associated infections. We also explored temporal relationships between site scores and common surrogate outcome measures such as total antibiotic consumption and CDIs.

All sites had existing AMS and IPC structures, though strategies and monitoring practices varied widely. The final tool, comprising 62 QIs grouped into thematic domains, was user-friendly and required minimal interpretation indicating clarity and objectivity. It also appeared sensitive to changes in program activities over time (e.g. during the COVID-19 pandemic). Sites in the highest quartile for AMS capacity generally demonstrated stable and declining antibiotic use, while lower-scoring sites showed numerically higher use over time—suggesting that strengthening of ‘weaker’ programs may require reaching a certain threshold before influencing prescribing behaviour.

Our findings align with previous studies showing that specific stewardship practices and strong contextual factors are associated with improved prescribing practices. An observational study conducted in Canadian hospitals found that only part of the 7-fold variation in antibiotic use was due to patient factors, with the remainder attributable to differences in resources and AMS practices, notably, audit-feedback and intravenous to oral conversion policies.^17^ A recent mixed-methods study conducted in USA linked lower antibiotic use to four contextual factors: access to stewardship guidance, strong pharmacist–physician collaboration, supportive infrastructure and highly engaged ID physicians who advocated for stewardship principles.^18^ Another study adapting a Quality Improvement (QI) framework (Three Antimicrobial Stewardship E’s, TASE) showed that sustained reductions in vancomycin and piperacillin–tazobactam use required continuous, repeated AMS efforts, rather than single interventions, with periods of high intensity activities (e.g. case-specific feedback, education, antibiotic algorithm updates, AMS policy changes) driving improvements.^19^

To our knowledge, few other studies have developed numerical scoring tools linking program performance with outcomes, or combine AMS and IPC assessments into a single tool providing a holistic site assessment. The CDC’s Global Antibiotic Stewardship Evaluation Tool (G-ASET) is comprehensive but resource-intensive and focuses on foundational AMS elements (e.g. strong leadership), giving less weight to specific interventions.^6^ Its presence/absence scoring system may overestimate programs with many checklist items while undervaluing those with fewer but higher-impact activities.

A recent study in England surveyed stewardship leads using a 30-item AMS score (0–3 per component) and explored potential associations with antibiotic use.^20^ No significant associations were found, though some trends emerged. For example, hospitals with lower scores showed higher baseline use of Watch antibiotics, the WHO AWaRe category that includes broader-spectrum agents with higher resistance potential.^21,22^ It is important to note that scores in that study reflected the *complexity* of program components, whereas our tool assigns higher scores to activities expected to have greater impact (e.g. audit-feedback).^16–18^

A 2013 study conducted in USA asked stewardship leads at academic hospitals to score program ‘intensity’ using 11 components grouped into ‘resources’ (e.g. stewardship pharmacists’ involvement) and ‘strategies’ (e.g. pre-authorization, post-prescription audit and feedback). Total AMS scores were not associated with antibiotic use but higher ‘strategy’ scores correlated with lower use of target antibiotics.^23^ In contrast, several studies using quantitative IPC scoring systems (e.g. WHO IPCAF score or a summary version focused on multimodal IPC strategies) have shown that higher IPC scores are associated with lower healthcare-associated infection rates.^24–26^

Our study has limitations.

First, the scoring system has not been formally validated and was developed in high-income tertiary hospitals with established IPC and AMS programs during a study period that included the COVID-19 pandemic and did not include MRSA-specific precautions, which may limit generalizability.

Second, CDIs were infrequent, making associations with IPC and AMS scores difficult to interpret. Since incidence of CDI reflects the combined influence of antibiotic exposure, IPC practices, patient case mix and environmental factors, the utility of CDI as a primary AMS effectiveness metric is limited in low-incidence settings.

Third, while monitoring antibiotic consumption is a pragmatic measure of AMS program impact, prescription appropriateness would be a better marker of program quality or effectiveness. Future studies would benefit from composite outcome frameworks that would integrate AMU, prescription quality and selected infection-related outcomes.

Finally, the expert panel was relatively small and familiar with one another, as all members participated in the PILGRIM study, which may have facilitated consensus more than in a group of experts who had no prior collaborations. We also did not formally appraise the quality of evidence supporting each quality indicator, although key indicators are unlikely to have been missed. Nonetheless, we propose a structured, holistic IPC and AMS scoring system that provides a useful measure of hospital capacity and allows adjustment for site-level factors. In a recent study, we showed that a five-factor patient score (including haematologic malignancies, immunosuppressive medication, past hospitalization and planned surgery) accurately predicted the likelihood of antibiotic treatment in newly hospitalized patients and may support AMS-focused clinical studies.^27^

Beyond identifying needs and tracking program performance over time, this IPC and AMS site assessment, potentially combined with a composite outcome framework that integrates antimicrobial use, process indicators, patient-specific predictive score and infection-related outcomes, can strengthen the methodological rigour of AMR-related clinical trials. If externally validated, this tool could support stratified randomization (e.g. high versus low scoring sites), tailored intervention and analysis of effect modifiers.

In summary, the PILGRIM site assessment for IPC and AMS is a concise, user-friendly tool that can standardize evaluations for acute-care hospitals and help assess readiness for AMR-related clinical trials. Preliminary data suggest that higher scores correlate with stronger programs and stable or declining antibiotic use, while lower scores reflect weaker programs with increasing antibiotic use. Future research is needed to evaluate reliability and to examine how individual program components relate to prescribing quality.

## Supplementary Material

dlag013_Supplementary_Data

## Transparency declarations

V.Z. holds an Antimicrobial Stewardship Fellowship Award from the McGill AMR Centre. M.S. holds operating grants from the Canadian Institutes for Health Research (CIHR) and reports being a member of the AMR Advisory Group for the Public Health Agency of Canada. M.J.G.T.V. declares to have received grants or contracts from MSD, Heel, BioNTech, Roche, SD Biosensor and Tillotts; she received consulting fees from Ferring, Tillotts, Bioaster and payment or honoraria for lectures from Akademie für Ärztliche Fort- und Weiterbildung, Akadmie für Infektionsmedizin, Astra Zeneca, bioMerieux, DGI, EUMEDICA, European Society of Neurogastroenterology, Falk Foundation, Ferring, FomF GmbH, Förderkreis Malteser, Frankfurter Bürger Universität, GILEAD, GSK, Helios Kliniken, Hessisches Landessozialgericht, Janssen Cilag GmbH, Jörg Eikerle Beratung, Klinikum Leverkusen, Lahn-Dill Kliniken, Landesärztekammer Hessen, LMU Kliniken, Med. Gesellschaft Bad Homburg, MSD, Pfizer, St. Vincent Hospital and Tillotts. Other authors do not have any conflicts of interest with relevance to this work.

## Supplementary data

Figures S1–S6 and Tables S1–S3 are available as Supplementary data at *JAC-AMR* Online.
