# Supplementary material for: Development of a quantitative self-assessment tool for hospital antimicrobial stewardship and infection control programs: a step towards standardizing clinical studies
Source: JAC Antimicrob Resist. 2026 Feb 6;8(1):dlag013. doi: 10.1093/jacamr/dlag013 (PMC12877872; doi:10.1093/jacamr/dlag013)

SUPPLEMENTARY MATERIAL

[Table 1S: Results of the consensus procedure for infection prevention and control (IPC) quality indicators 2](#_Toc217387554)

[Table 2S: Results of the consensus procedure for antimicrobial stewardship programs (AMS) quality indicators 5](#_Toc217387555)

[Table 3S: Infection prevention and control (IPC) self-assessment scores by site 7](#_Toc217387556)

[Figure 1S: Total antimicrobial stewardship programs (AMS) scores per year for each site 10](#_Toc217387557)

[Figure 2S: Association between total antibiotic use and antimicrobial stewardship score for each site 11](#_Toc217387558)

[Figure 3S: Association between total antibiotic use and stewardship “Interventions” score 12](#_Toc217387559)

[Figure 4S: Association between incidence of *C difficile* infections and scores 13](#_Toc217387560)

[Figure 5S: Infection and Prevention Control (IPC) scores and hand disinfectant consumption 15](#_Toc217387561)

[Figure 6S: Hand Hygiene scores versus total Infection Prevention and Control scores 16](#_Toc217387562)

### Table 1S: Results of the consensus procedure for infection prevention and control (IPC) quality indicators

| Quality indicator | Domain | First round | Second round | Third round | | | | Final decision |
| --- | --- | --- | --- | --- | --- | --- | --- | --- |
|  |  |  |  | Proposed score | | | Consensus meeting |  |
|  |  |  |  | Yes | Partial | No |  |  |
| 1. ~~Number of single rooms; double or more occupancy rooms~~   Are all patient rooms single occupancy? | Structure | Disagreement | Rephrased | 2  >50% rooms | 1  10-49% | 0  <10% | Score Accepted | IPC QI1 |
| 1. Is every patient room equipped with one bathroom including sink, toilet and shower? | Structure | QI Accepted | ND | 2  all rooms | 1  some rooms/ all rooms partially equipped | 0 | Score Accepted | IPC QI2 |
| 1. Are disinfectant dispensers available in each patient room? | Structure | QI Accepted | ND | 2  all rooms | 1  some rooms | 0 | Score Accepted | IPC QI3 |
| 1. Full time equivalents (FTE): Nurses | Structure | QI Accepted | ND | 2  ≥1:5 | 1  1:6 | 0  <1:6 | Score Accepted | IPC QI4 |
| 1. Full time equivalents Number of physicians including residents and attending staff who work at the ward) | Structure | Disagreement | Rejected |  |  |  |  |  |
| Pathogen: VRE |  |  |  |  |  |  |  |  |
| 1. Do you regularly screen patients for VRE carriage? | Precautions | Disagreement | Accepted | 2  all | 1  only those with certain criteria | 0 | Score Accepted | IPC QI5 |
| 1. Single room contact precautions applied? | Precautions | QI Accepted | ND | 2  all | 1  cohorting | 0 | Disagreement, changed to:  2= all patients  1= some patients  0= no | IPC QI6 |
| 1. Wearing of gloves when entering the room | Precautions | Disagreement | Rejected |  |  |  |  |  |
| 1. Wearing of gowns when entering the room | Precautions | Disagreement | Rejected |  |  |  |  |  |
| 1. Wearing of hair cover when entering the room | Precautions | Disagreement | Rejected |  |  |  |  |  |
| 1. ~~Wearing of gloves before direct patient contact~~   Do you wear gloves AND gowns before direct patient contact? | Precautions | Disagreement | Combined and rephrased |  | 1  gloves and gowns | 0  gowns only OR none | Score Accepted | IPC QI7 |
| 1. ~~Wearing of gowns before direct patient contact~~ | Precautions | Disagreement |  |  |  |  |  |  |
| 1. Wearing of hair cover before direct patient contact | Precautions | Disagreement | Rejected |  |  |  |  |  |
| 1. Wearing of gloves before patients leave the room | Precautions | Disagreement | Rejected |  |  |  |  |  |
| 1. Wearing of gowns before patients leave the room | Precautions | Disagreement | Rejected |  |  |  |  |  |
| 1. Wearing of hair cover before patients leave the room | Precautions | Disagreement | Rejected |  |  |  |  |  |
| Pathogen: EPE |  |  |  |  |  |  |  |  |
| 1. Do you regularly screen patients for EPE carriage? | Precautions | Disagreement | Accepted | 2  all | 1  those meeting certain criteria | 0 | Score Accepted | IPC QI8 |
| 1. Single room contact precautions applied? | Precautions | QI Accepted | ND | 2  all | 1  cohorting | 0 | Disagreement & changed to:  2= all patients  1= some patients  0= no | IPC QI9 |
| 1. Wearing of gloves when entering the room | Precautions | Disagreement | Rejected |  |  |  |  |  |
| 1. Wearing of gowns when entering the room | Precautions | Disagreement | Rejected |  |  |  |  |  |
| 1. Wearing of hair cover when entering the room | Precautions | Disagreement | Rejected |  |  |  |  |  |
| 1. ~~Wearing of gloves before direct patient contact~~   Do you wear gloves AND gowns before direct patient contact? | Precautions | Disagreement | Combined and rephrased |  | 1  gloves and gowns | 0  gown only OR none | Score Accepted | IPC QI10 |
| 1. ~~Wearing of gowns before direct patient contact~~ | Precautions | Disagreement |  |  |  |  |  |  |
| 1. Wearing of hair cover before direct patient contact | Precautions | Disagreement | Rejected |  |  |  |  |  |
| 1. Wearing of gloves before patients leave the room | Precautions | Disagreement | Rejected |  |  |  |  |  |
| 1. Wearing of gowns before patients leave the room | Precautions | Disagreement | Rejected |  |  |  |  |  |
| 1. Wearing of hair cover before patients leave the room | Precautions | Disagreement | Rejected |  |  |  |  |  |
| Pathogen: CRE/CPE |  |  |  |  |  |  |  |  |
| 1. Do you regularly screen patients for CRE/CPE carriage? | Precautions | QI Accepted | ND | 2  all | 1  only those meeting certain criteria | 0 | Disagreement & changed to:  2= all patients  1= some patients  0= no | IPC QI11 |
| 1. Single room contact precautions applied? | Precautions | QI Accepted | ND | 2 | 1 | 0 |  | IPC QI12 |
| 1. Wearing of gloves when entering the room | Precautions | Disagreement | Rejected |  |  |  |  |  |
| 1. Wearing of gowns when entering the room | Precautions | Disagreement | Rejected |  |  |  |  |  |
| 1. Wearing of hair cover when entering the room | Precautions | Disagreement | Rejected |  |  |  |  |  |
| 1. ~~Wearing of gloves before direct patient contact~~   Do you wear gloves AND gowns before direct patient contact? | Precautions | Disagreement | Combined and rephrased |  | 1  gloves and gowns | 0  gown only OR none | Score Accepted | IPC QI13 |
| 1. ~~Wearing of gowns before direct patient contact~~ | Precautions | Disagreement |  |  |  |  |  |  |
| 1. Wearing of hair cover before direct patient contact | Precautions | Disagreement | Rejected |  |  |  |  |  |
| 1. Wearing of gloves before patients leave the room | Precautions | Disagreement | Rejected |  |  |  |  |  |
| 1. Wearing of gowns before patients leave the room | Precautions | Disagreement | Rejected |  |  |  |  |  |
| 1. Wearing of hair cover before patients leave the room | Precautions | Disagreement | Rejected |  |  |  |  |  |
| Pathogen: C.diff |  |  |  |  |  |  |  |  |
| 1. Single room contact precautions applied? | Precautions | QI Accepted | ND | 2  all | 1  cohorting | 0 | Disagreement & changed to:  2= all patients  1= some patients  0= no | IPC QI14 |
| 1. Wearing of gloves when entering the room | Precautions | Disagreement | Rejected |  |  |  |  |  |
| 1. Wearing of gowns when entering the room | Precautions | Disagreement | Rejected |  |  |  |  |  |
| 1. Wearing of hair cover when entering the room | Precautions | Disagreement | Rejected |  |  |  |  |  |
| 1. ~~Wearing of gloves before direct patient contact~~   Do you wear gloves AND gowns before direct patient contact? | Precautions | Disagreement | Combined and rephrased |  | 1  gloves and gowns | 0  gown only OR none | Score Accepted | IPC QI15 |
| 1. ~~Wearing of gowns before direct patient contact~~ | Precautions | Disagreement |  |  |  |  |  |  |
| 1. Wearing of hair cover before direct patient contact | Precautions | Disagreement | Rejected |  |  |  |  |  |
| 1. Wearing of gloves before patients leave the room | Precautions | Disagreement | Rejected |  |  |  |  |  |
| 1. Wearing of gowns before patients leave the room | Precautions | Disagreement | Rejected |  |  |  |  |  |
| 1. Wearing of hair cover before patients leave the room | Precautions | Disagreement | Rejected |  |  |  |  |  |
| 1. Frequency of cleaning of high touch objects in patient room | Cleaning | QI Accepted | ND | 2  twice a day | 1  once daily | 0  if necessary/ on demand | Score Accepted | IPC QI16 |
| 1. Frequency of cleaning of other objects/spots in patient room | Cleaning | QI Accepted | ND | 2  twice a day | 1  once daily | 0  if necessary/ on demand | Score Accepted | IPC QI17 |
| 1. Cleaning of spots close to patient bed performed by nurses or cleaning staff. | Cleaning | QI Accepted | ND | 2  by cleaning staff | 1  By nurses | 0 | Score Accepted | IPC QI18 |
| 1. Are there cleaning standards available for the cleaning of rooms after patient discharge, if the patients were NOT colonized or infected with a multi-resistant pathogen | Cleaning | QI Accepted | ND | 2  proper disinfectant | 1  without proper disinfectant | 0 | Score Accepted | IPC QI19 |
| 1. Are there cleaning standards available for the cleaning of rooms after patient discharge, if the patients WERE colonized or infected with a multi-resistant pathogen/C. difficile? | Cleaning | QI Accepted | ND | 2  proper disinfectant | 1  without proper disinfectant | 0 | Score Accepted | IPC QI20 |
| 1. Are there routinely performed cleaning controls by the hygiene department or the cleaning company? | Cleaning | QI Accepted | ND | 2 |  | 0 | Score Accepted | IPC QI21 |
| 1. Are there training courses for the cleaning staff provided by the hygiene department or the cleaning company? | Cleaning | QI Accepted | ND | 2 |  | 0 | Score Accepted | IPC QI22 |
| 1. Is hand disinfectant consumption (ml/patient day) assessed for this department? | Cleaning | QI Accepted | ND | 2 |  | 0 | Score Accepted | IPC QI23 |
| 1. Is there clear visible information on hand hygiene procedures (e.g. poster on “5 Moments for hand hygiene” of the WHO) in at least three important/visible points at the hospital ward? | Hand Hygiene | QI Accepted | ND | 2 |  | 0 | Score Accepted | IPC QI24 |
| 1. Are there regularly provided trainings on hand hygiene procedures for healthcare professionals? | Hand Hygiene | QI Accepted | ND | 2 |  | 0 | Score Accepted | IPC QI25 |
| 1. Are there regularly performed (at least once per year) audits of hand hygiene compliance? | Hand Hygiene | QI Accepted | ND | 2 |  | 0 | Score Accepted | IPC QI26 |
| 1. Is feedback provided to hospital staff after evaluation of hand hygiene compliance? | Hand Hygiene | QI Accepted | ND | 2 |  | 0 | Score Accepted | IPC QI27 |

Strikethrough and italic texts show the rephrasing process of the quality indicators.

ND, not discussed; IPC, Infection prevention and control; QI, quality indicator.

### Table 2S: Results of the consensus procedure for antimicrobial stewardship programs (AMS) quality indicators

| Quality indicators | Domain | First round | Second round | Third round | | | | Final decision |
| --- | --- | --- | --- | --- | --- | --- | --- | --- |
|  |  |  |  | Proposed score | | | Consensus meeting |  |
|  |  |  |  | Yes | Partial | No |  |  |
| 1. Does your facility have a formal, written statement of support from leadership that supports efforts to improve antibiotic use? | Structure | QI Accepted | ND | 1 |  | 0 | Score Accepted | AMS QI01 |
| 1. Does your facility receive any budgeted financial support for antibiotic stewardship activities (e.g. support for salary, training, or IT support)? | Structure | QI Accepted | ND | 1 |  | 0 | Score Accepted | AMS QI02 |
| 1. Is there a physician leader responsible for program outcomes of stewardship activities at your facility? | Structure | QI Accepted | ND | 1 |  | 0 | Score Accepted | AMS QI03 |
| 1. Is there a pharmacist leader responsible for working to improve antibiotic use at your facility? | Structure | QI Accepted | ND | 1 |  | 0 | Score Accepted | AMS QI04 |
| Does any of the staff below work with the stewardship leaders to improve antibiotic use? |  |  |  |  |  |  |  |  |
| 1. Clinicians | Structure | QI Accepted | ND | 1 |  | 0 | Score Accepted | AMS QI05 |
| 1. Microbiology (Laboratory) | Structure | QI Accepted | ND | 1 |  | 0 | Score Accepted | AMS QI06 |
| 1. Any of IPC, Information Technology (IT), Nursing, QI, other | Structure | QI Accepted | ND | 1 |  | 0 | Score Accepted | AMS QI07 |
| 1. Does your facility have a policy that requires prescribers to document in the medical record or during order entry a dose, duration, and indication for all antibiotic prescriptions? | Guideline/  policy | QI Accepted | ND | 2 | 1 | 0 | Score Accepted | AMS QI08 |
| 1. Does your facility have facility-specific treatment recommendations, based on national guidelines and local susceptibility, to assist with antibiotic selection for common clinical conditions? | Guideline/  policy | QI Accepted | ND | 2 | 1 | 0 | Score Accepted | AMS QI09 |
| 1. Is there a formal procedure for all clinicians to review the appropriateness of all antibiotics 48 hours after the initial orders (e.g. antibiotic time out)? | Guideline/  policy | QI Accepted | ND | 2 | 1 | 0 | Score Accepted | AMS QI10 |
| 1. Do specified antibiotic agents need to be approved by a physician or pharmacist prior to dispensing (i.e., pre-authorization) at your facility? | Guideline/  policy | QI Accepted | ND | 1 |  | 0 | Score Accepted | AMS QI11 |
| 1. Does a physician or pharmacist review courses of therapy for specified antibiotic agents (i.e., prospective audit with feedback) at your facility? | Interventions | QI Accepted | ND | 3 | 2 or 1 | 0 | Score Accepted | AMS QI12 |
| 1. Automatic changes from intravenous to oral antibiotic therapy in appropriate situations? | Interventions | QI Accepted | ND | 1 |  | 0 | Score Accepted | AMS QI13 |
| 1. Dose adjustments in cases of organ dysfunction? | Interventions | QI Accepted | ND | 1 |  | 0 | Score Accepted | AMS QI14 |
| 1. Dose optimization (pharmacokinetics/pharmacodynamics) to optimize the treatment of organisms with reduced susceptibility? | Interventions | QI Accepted | ND | 1 |  | 0 | Score Accepted | AMS QI15 |
| 1. Automatic alerts in situations where therapy might be unnecessarily duplicative? | Interventions | QI Accepted | ND | 1 |  | 0 | Score Accepted | AMS QI16 |
| 1. Time-sensitive automatic stop orders for specified antibiotic prescriptions? | Interventions | Disagreement | Rejected (duplicate with no. 10) |  |  |  |  |  |
| Specific interventions to ensure optimal treatment of: |  |  |  |  |  |  |  |  |
| 1. Community-acquired pneumonia (CAP) | Interventions | QI Accepted | ND | 1 |  | 0 | Score Accepted | AMS QI17 |
| 1. Urinary tract infections (UTI) | Interventions | QI Accepted | ND | 1 |  | 0 | Score Accepted | AMS QI18 |
| 1. Skin and soft tissue infections | Interventions | QI Accepted | ND | 1 |  | 0 | Score Accepted | AMS QI19 |
| 1. Surgical prophylaxis | Interventions | QI Accepted | ND | 1 |  | 0 | Score Accepted | AMS QI20 |
| 1. Empiric treatment of Methicillin-resistant Staphylococcus aureus (MRSA) | Interventions | QI Accepted | ND | 1 |  | 0 | Score Accepted | AMS QI21 |
| 1. Non-C. difficile Infection (CDI) antibiotics in new cases of CDI (eg. Review of all CDI to stop unnecessary antibiotics) | Interventions | QI Accepted | ND | 1 |  | 0 | Score Accepted | AMS QI22 |
| 1. Culture-proven invasive (e.g. blood stream) infections | Interventions | QI Accepted | ND | 1 |  | 0 | Score Accepted | AMS QI23 |
| 1. Does your stewardship program monitor adherence to a documentation policy (dose, duration, and indication)? | Monitoring | QI Accepted | ND | 2 | 1 | 0 | Score Accepted | AMS QI24 |
| 1. Does your stewardship program monitor adherence to facility-specific treatment recommendations? | Monitoring | QI Accepted | ND | 2 | 1 | 0 | Score Accepted | AMS QI25 |
| 1. Does your stewardship program monitor compliance with one or more of the specific interventions in place? | Monitoring | QI Accepted | ND | 2 | 1 | 0 | Score Accepted | AMS QI26 |
| 1. Does your facility track rates of C. difficile infection? | Surveillance | QI Accepted | ND | 2 |  | 0 | Score Accepted | AMS QI27 |
| 1. Does your facility monitor antibiotic use (consumption) by counts of antibiotic(s) administered to patients per day (Days of Therapy; DOT) | Surveillance | Disagreement | Combined and rephrased | 2  (by DOT or DDD) | 1  (by expenditure only) | 0 | Score Accepted | AMS QI28 |
| 1. Does your facility monitor antibiotic use (consumption) by defined daily dose (DDD)? | Surveillance | Disagreement |  |  |  |  |  |  |
| 1. Does your facility monitor antibiotic use (consumption) by direct expenditure for antibiotics (purchasing costs)? | Surveillance | Disagreement |  |  |  |  |  |  |
| 1. Does your facility monitor antibiotic use (consumption) At the unit level | Surveillance | QI Accepted | ND | 2  (annually) | 1  (<annually) | 0 | Score Accepted | AMS QI29 |
| 1. Does your facility monitor antibiotic use (consumption) at the facility wide level | Surveillance | QI Accepted | ND | 2  (annually) | 1  (<annually) | 0 | Score Accepted | AMS QI30 |
| 1. Does your facility produce an antibiogram (cumulative antibiotic susceptibility report) ? | Reporting/  Education/  training | QI Accepted | ND | 2  (annually) | 1  (<annually) | 0 | Score Accepted | AMS QI31 |
| 1. Does your stewardship program share facility-specific reports on antibiotic use with prescribers? | Reporting/  Education/  training | QI Accepted | ND | 2  (annually) | 1  (<annually) | 0 | Score Accepted | AMS QI32 |
| 1. Has a current antibiogram been distributed to prescribers at your facility? | Reporting/  Education/  training | QI Accepted | ND | 1 |  | 0 | Score Accepted | AMS QI33 |
| 1. Do prescribers ever receive direct, personalized communication about how they can improve their antibiotic prescribing? | Reporting/  Education/  training | QI Accepted | ND | 2  (immediate feedback) | 1  (aggregate or  for some indications/  antibiotics) | 0 | Score Accepted | AMS QI34 |
| 1. Does your stewardship program provide education to clinicians and other relevant staff on improving antibiotic prescribing? | Reporting/  Education/  training | QI Accepted | ND | 1 |  | 0 | Score Accepted | AMS QI35 |

ND, not discussed; QI, quality indicator; AMS, antimicrobial stewardship program.

### Table 3S: Infection prevention and control (IPC) self-assessment scores by site

| Site | Year | Indicator department | IPC STRUCTURE score  max: 8 | IPC PRECAUTIONS score  max: 18 | IPC CLEANING score  max: 16 | IPC HAND HYGIENE score  max: 8 | IPC TOTAL SCORE max: 50 |
| --- | --- | --- | --- | --- | --- | --- | --- |
| S1 | 2019 | ID1 | 7 | 13 | 13 | 6 | 39 |
|  |  | ID2 | 7 | 13 | 13 | 6 | 39 |
|  | 2020 | ID1 | 7 | 13 | 13 | 6 | 39 |
|  |  | ID2 | 7 | 13 | 13 | 6 | 39 |
|  | 2021 | ID1 | 7 | 13 | 11 | 6 | 37 |
|  |  | ID2 | 7 | 13 | 11 | 6 | 37 |
|  | 2024 | Hospital | 7 | 13 | 14 | 6 | 40 |
| S2 | 2019 | ID1 | 7 | 14 | 11 | 3 | 35 |
|  |  | ID2 | 7 | 14 | 11 | 3 | 35 |
|  |  | ID3 | 7 | 14 | 11 | 3 | 35 |
|  | 2020 | ID1 | 7 | 14 | 12 | 3 | 36 |
|  |  | ID2 | 7 | 14 | 12 | 3 | 36 |
|  |  | ID3 | 7 | 14 | 12 | 3 | 36 |
|  | 2021 | ID1 | 7 | 14 | 12 | 3 | 36 |
|  |  | ID2 | 7 | 14 | 12 | 3 | 36 |
|  |  | ID3 | 7 | 14 | 12 | 3 | 36 |
|  | 2024 | Hospital | 6 | 13 | 15 | 4 | 38 |
| S3 | 2019 | ID1 | 7 | 14 | 13 | 2 | 36 |
|  |  | ID2 | 7 | 14 | 13 | 4 | 38 |
|  |  | ID3 | 7 | 14 | 13 | 2 | 36 |
|  | 2020 | ID1 | 7 | 14 | 13 | 2 | 36 |
|  |  | ID2 | 7 | 14 | 13 | 3 | 37 |
|  |  | ID3 | 7 | 14 | 13 | 2 | 36 |
|  | 2021 | ID1 | 7 | 14 | 13 | 4 | 38 |
|  |  | ID2 | 7 | 13 | 14 | 4 | 38 |
|  |  | ID3 | 7 | 14 | 13 | 2 | 36 |
|  | 2024 | Hospital | 7 | 14 | 11 | 8 | 40 |
| S4 | 2022 | ID1 | 6 | 11 | 13 | 2 | 32 |
|  |  | ID2 | 7 | 14 | 12 | 2 | 36 |
|  | 2024 | ID1 | 6 | 11 | 10 | 3 | 30 |
|  |  | ID2 | 7 | 14 | 10 | 3 | 34 |
| S5 | 2019-2022 | ID1 | 5 | 10 | 13 | 5 | 33 |
|  |  | ID2 | 5 | 10 | 13 | 5 | 33 |
|  |  | ID3 | 5 | 10 | 13 | 5 | 33 |
|  |  | ID4 | 6 | 10 | 13 | 5 | 34 |
|  | 2024 | Hospital | 3 | 10 | 12 | 5 | 30 |
| S6 | 2019 | ID1 | 7 | 12 | 14 | 8 | 41 |
|  |  | ID2 | 8 | 12 | 14 | 8 | 42 |
|  |  | ID3 | 6 | 12 | 14 | 8 | 40 |
|  | 2020-2021 | ID1 | 7 | 15 | 14 | 8 | 44 |
|  |  | ID2 | 8 | 15 | 14 | 8 | 45 |
|  |  | ID3 | 6 | 15 | 14 | 8 | 43 |
|  | 2024 | Hospital | 5 | 15 | 14 | 8 | 42 |
| S7 | 2019 | ID1 | 7 | 13 | 10 | 4 | 34 |
|  | 2020 | ID2 | 7 | 11 | 10 | 4 | 32 |
|  | 2024 | Hospital | 7 | 13 | 10 | 8 | 38 |
| S8 | 2019 | ID1 | 4 | 14 | 11 | 5 | 34 |
|  |  | ID2 | 8 | 14 | 9 | 1 | 32 |
|  | 2020 | ID1 | 4 | 14 | 9 | 5 | 32 |
|  |  | ID2 | 8 | 14 | 7 | 1 | 30 |
|  | 2024 | Hospital | 6 | 13 | 11 | 4 | 34 |

Table 4S: Antimicrobial stewardship program (AMS) self-assessment scores by site

| Site | Year | AMS Structure score max:7 | AMS Guideline/  policy score  max: 7 | AMS Interventions score max:14 | AMS Monitoring/  Surveillance   Score   max:14 | AMS Reporting/  education  score  max:8 | AMS TOTAL SCORE max:50 |
| --- | --- | --- | --- | --- | --- | --- | --- |
| S1 | 2019 | 6 | 2 | 7 | 8 | 4 | 27 |
|  | 2020 | 7 | 3 | 8 | 8 | 4 | 30 |
|  | 2021 | 7 | 4 | 8 | 9 | 6 | 34 |
|  | 2024 | 7 | 4 | 9 | 11 | 6 | 37 |
| S2 | 2019 | 6 | 3 | 1 | 8 | 6 | 24 |
|  | 2020 | 6 | 3 | 1 | 8 | 6 | 24 |
|  | 2021 | 6 | 3 | 1 | 8 | 6 | 24 |
|  | 2024 | 7 | 4 | 3 | 9 | 8 | 31 |
| S3 | 2019 | 7 | 5 | 1 | 9 | 7 | 29 |
|  | 2020 | 7 | 5 | 1 | 9 | 7 | 29 |
|  | 2021 | 7 | 5 | 1 | 9 | 7 | 29 |
|  | 2024 | 7 | 5 | 2 | 9 | 7 | 30 |
| S4 | 2022 | 5 | 3 | 2 | 8 | 5 | 23 |
|  | 2024 | 7 | 3 | 5 | 8 | 4 | 27 |
| S5 | 2019-2022 | 5 | 5 | 4 | 9 | 5 | 28 |
|  | 2024 | 4 | 5 | 2 | 8 | 5 | 24 |
| S6 | 2019 | 7 | 3 | 2 | 10 | 7 | 29 |
|  | 2020-2021 | 7 | 3 | 2 | 12 | 7 | 31 |
|  | 2024 | 7 | 5 | 3 | 12 | 8 | 35 |
| S7 | 2019 | 7 | 4 | 8 | 10 | 5 | 34 |
|  | 2020 | 7 | 4 | 8 | 10 | 5 | 34 |
|  | 2024 | 7 | 4 | 9 | 9 | 5 | 34 |
| S8 | 2019 | 7 | 5 | 3 | 10 | 8 | 33 |
|  | 2020 | 7 | 5 | 3 | 10 | 8 | 33 |
|  | 2024 | 7 | 5 | 3 | 10 | 8 | 33 |

### Figure 1S: Total antimicrobial stewardship programs (AMS) scores per year for each site


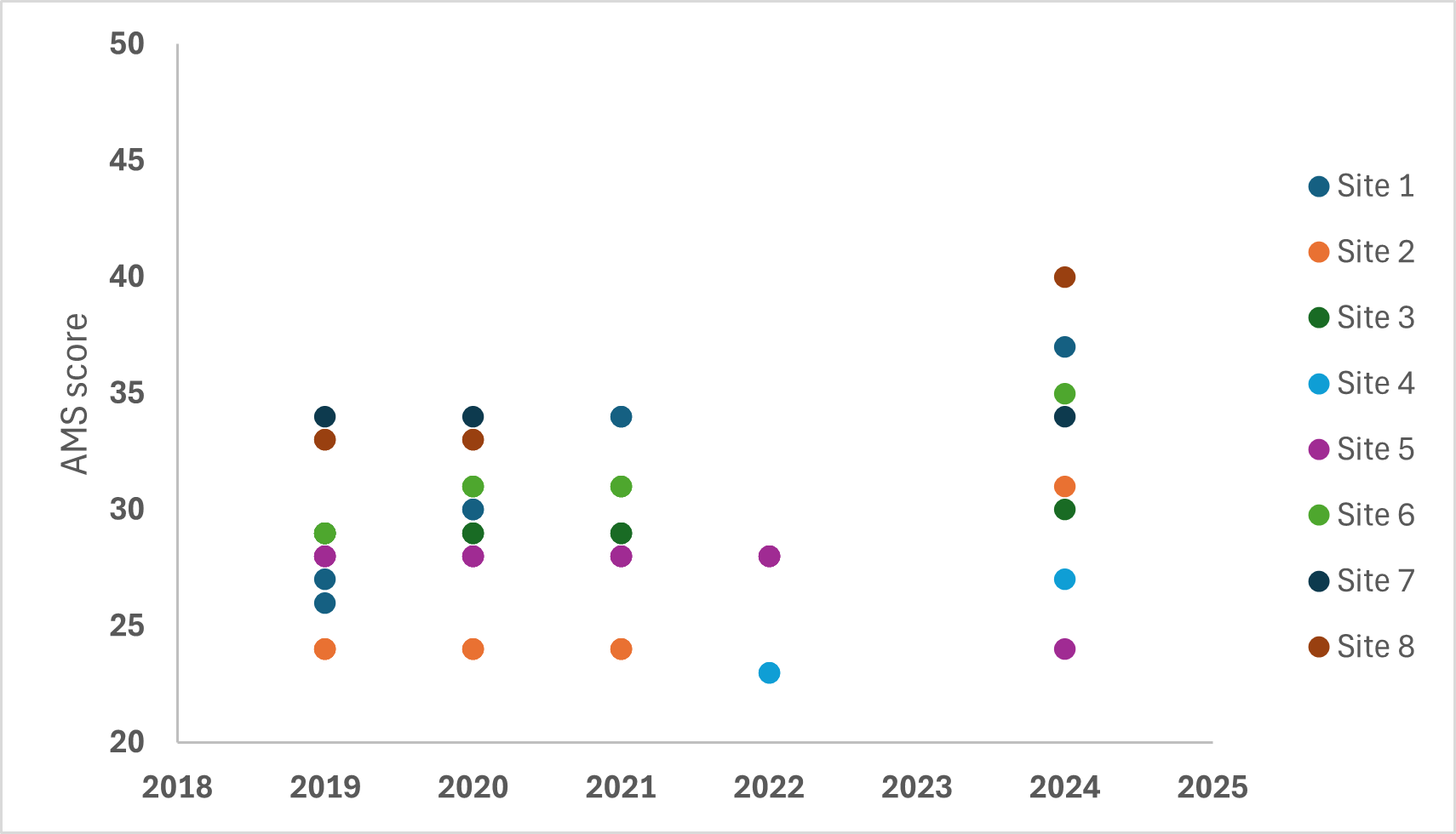


### Figure 2S: Association between total antibiotic use and antimicrobial stewardship score for each site


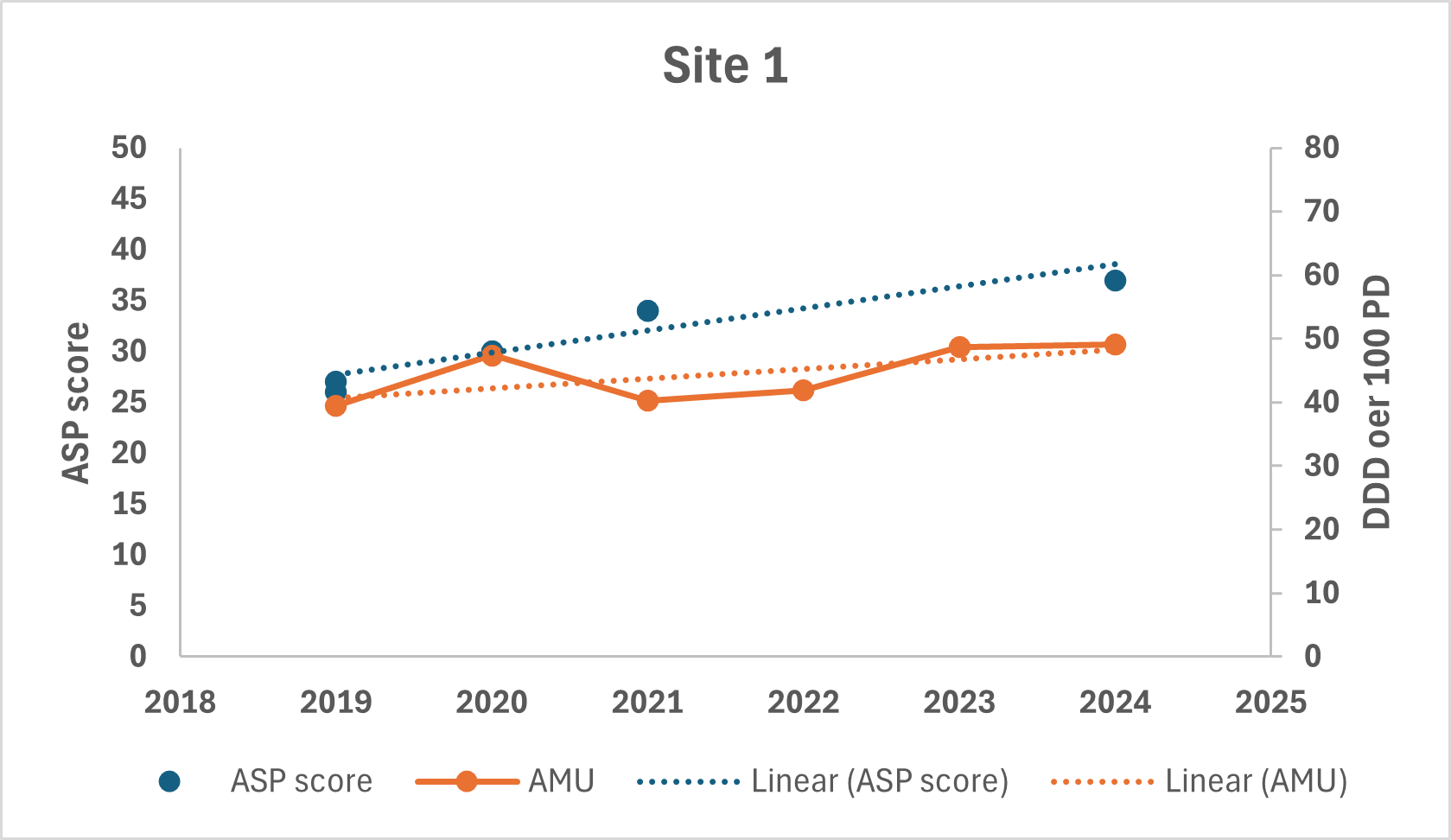

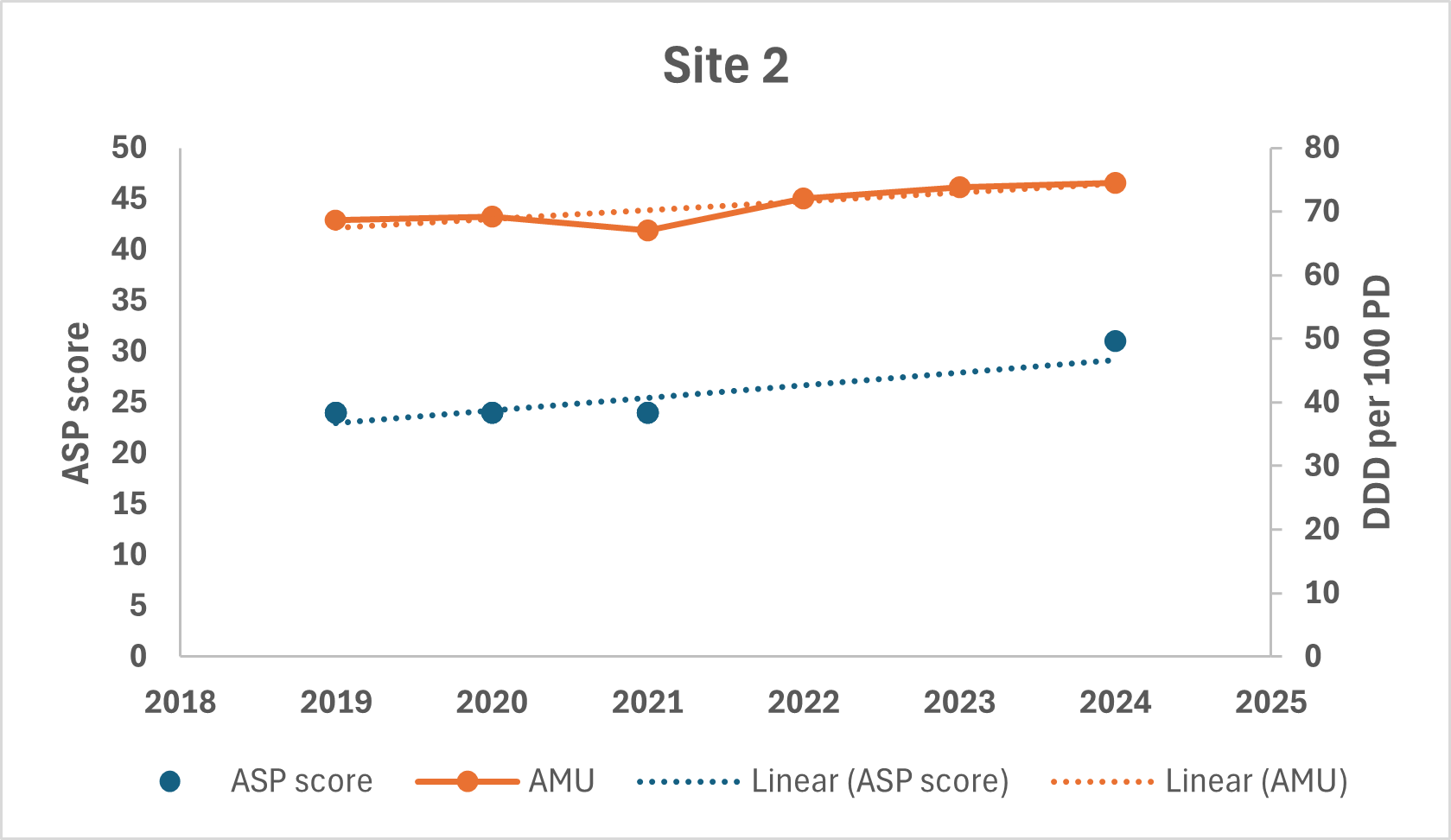


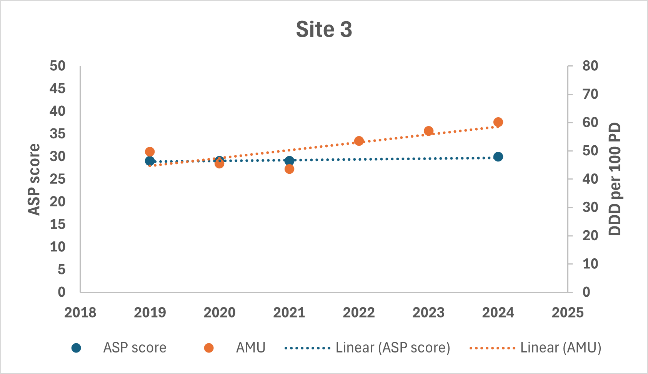

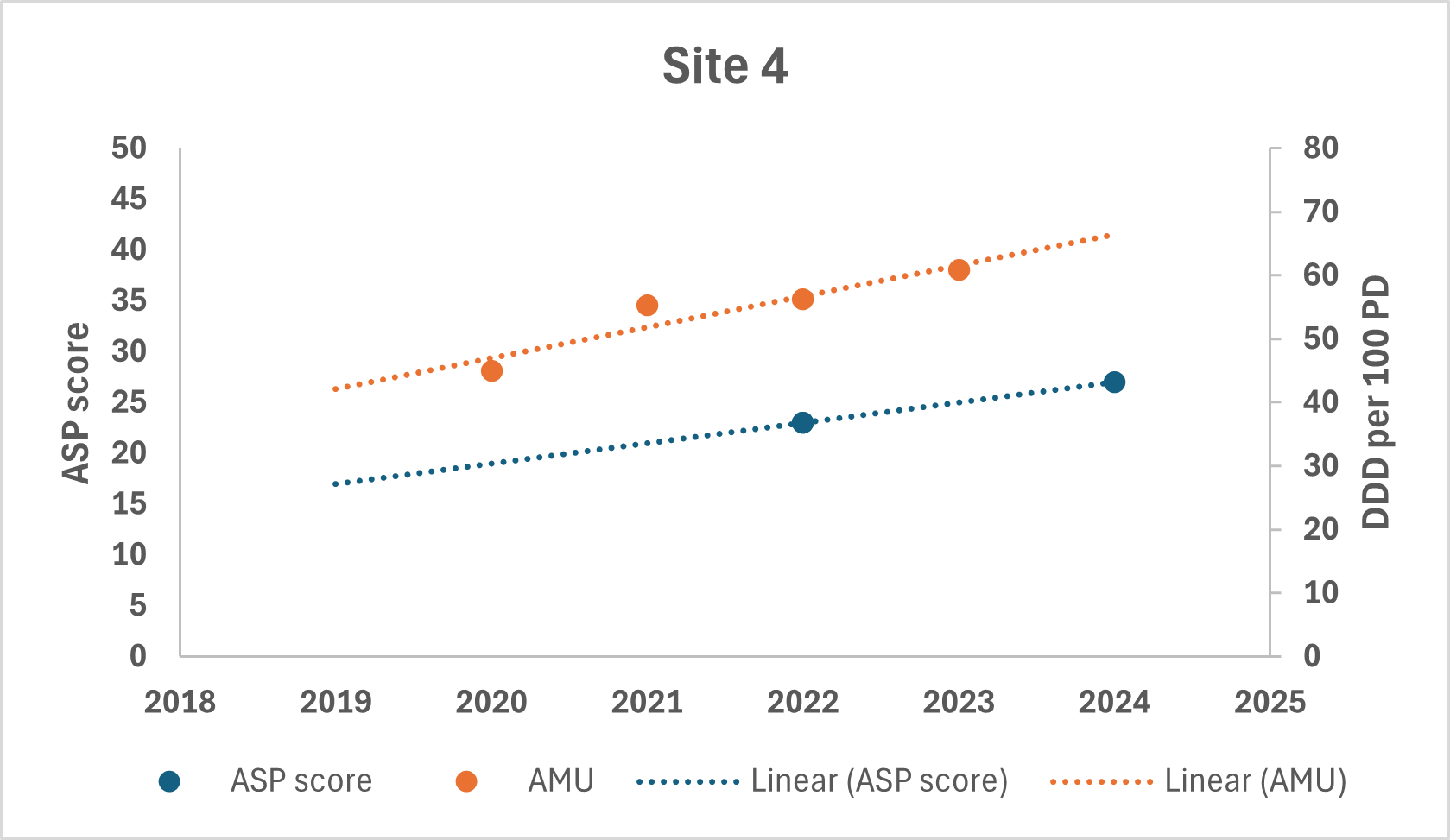


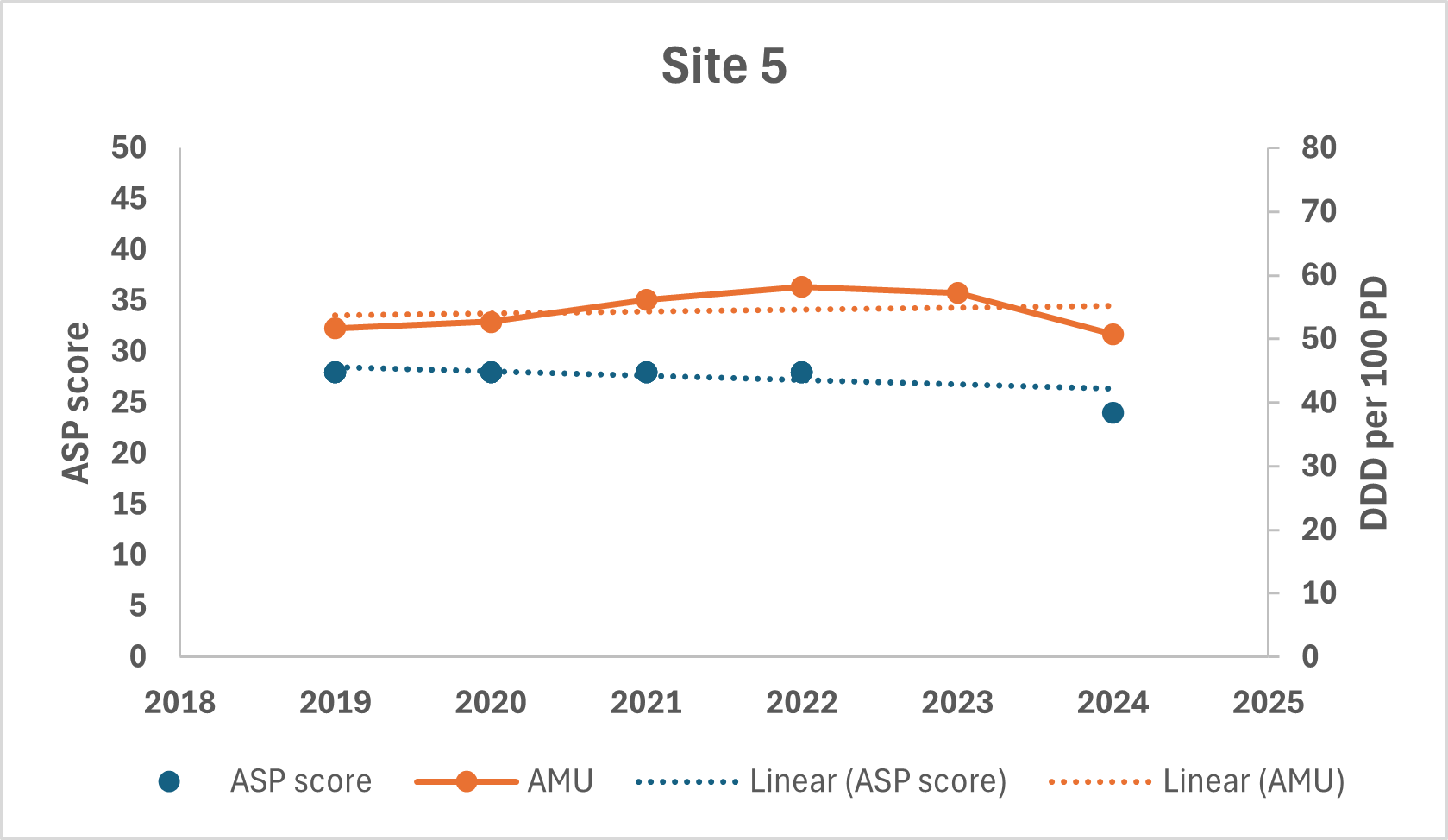

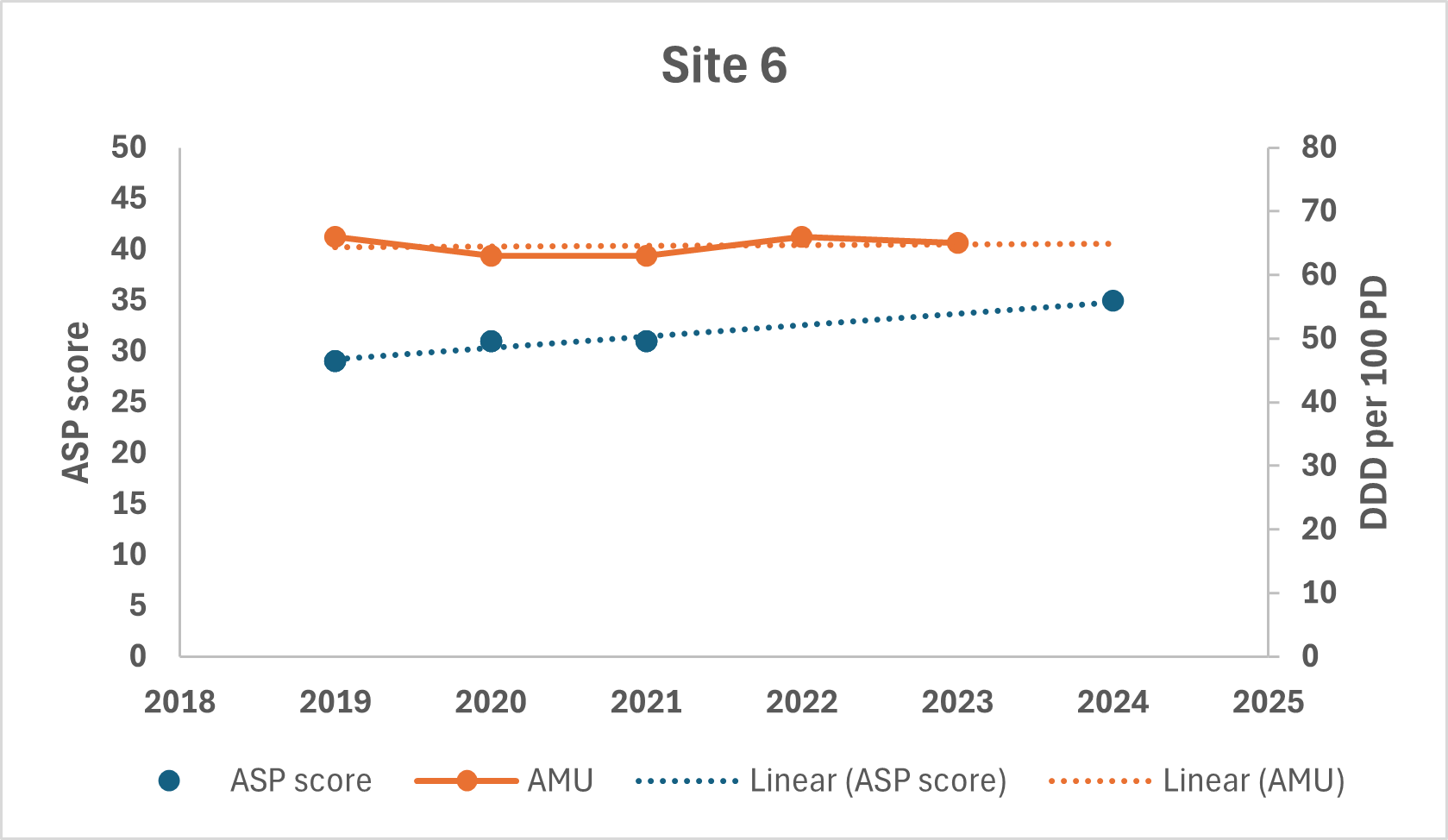


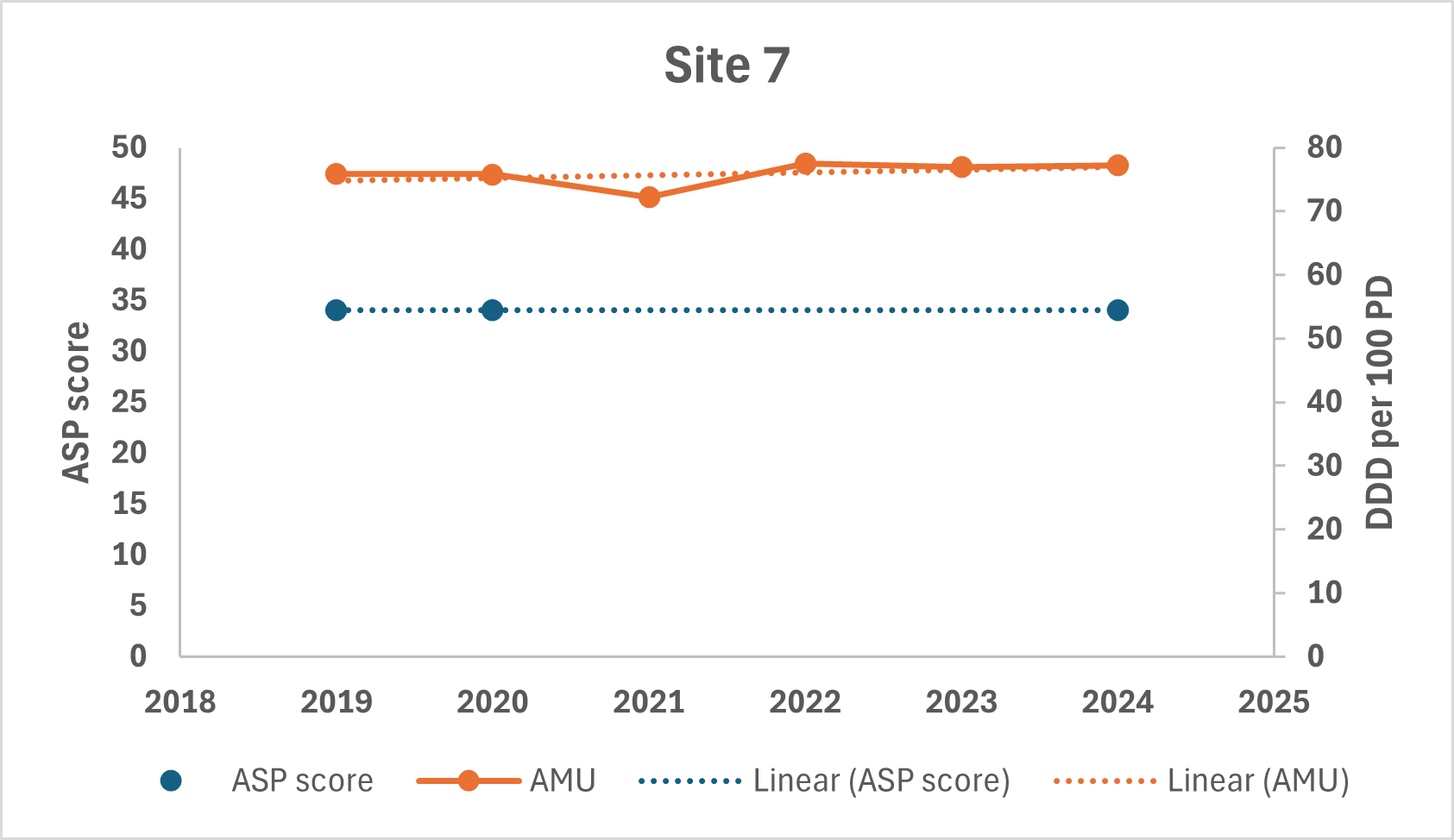

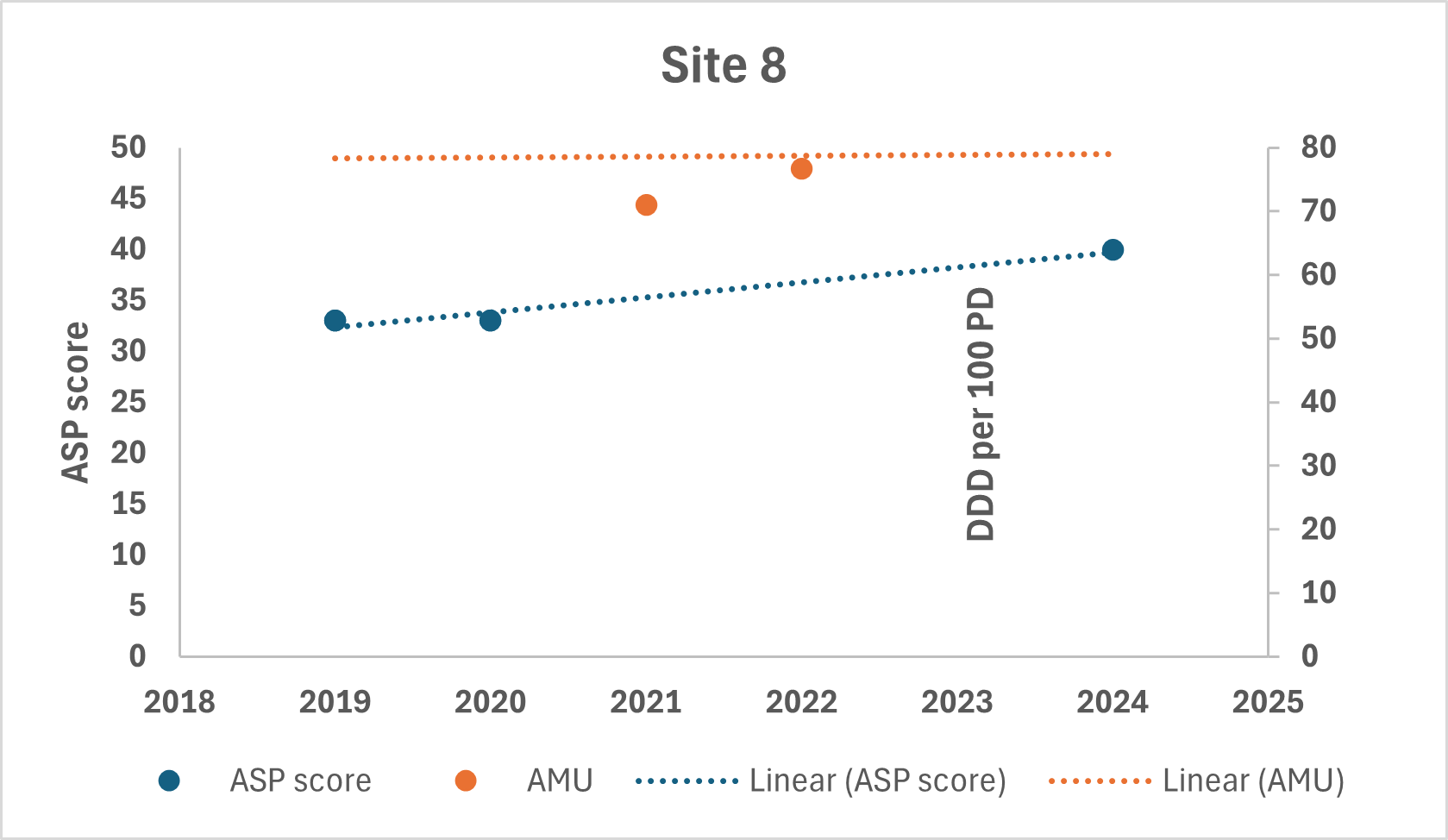


AMU: total antibiotic use; ASP score: antimicrobial stewardship site assessment score

### Figure 3S: Association between total antibiotic use and stewardship “Interventions” score


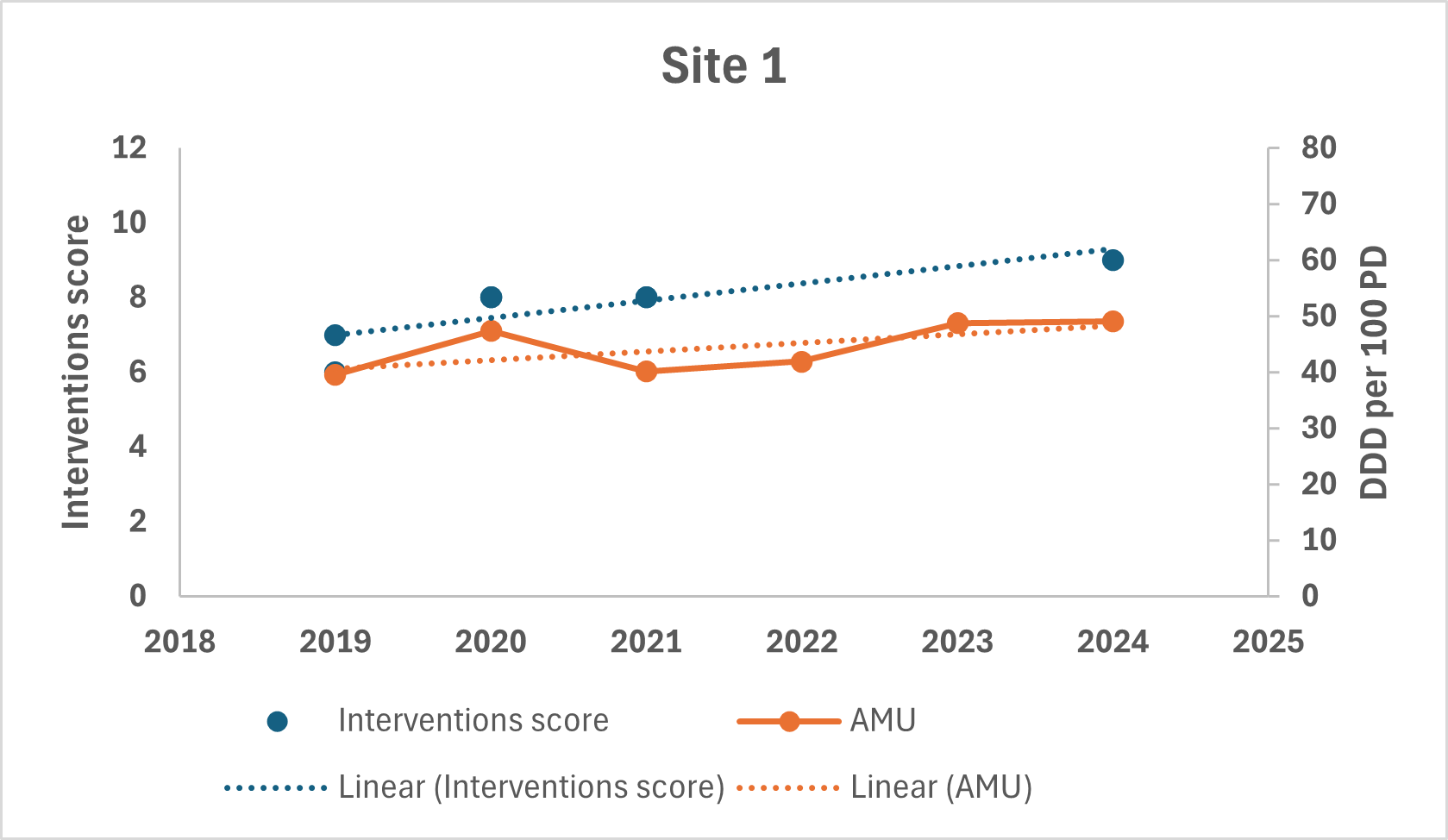

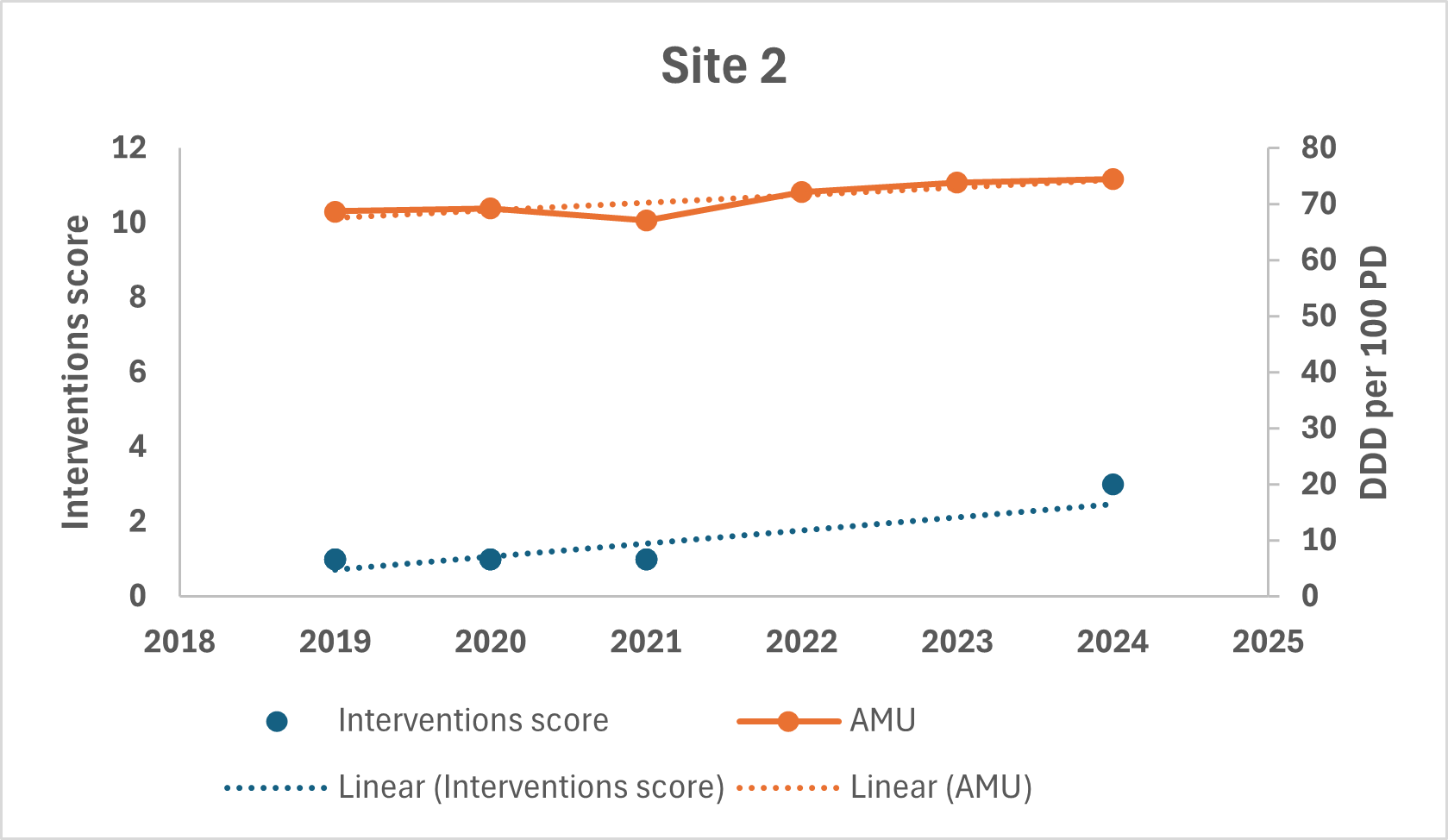


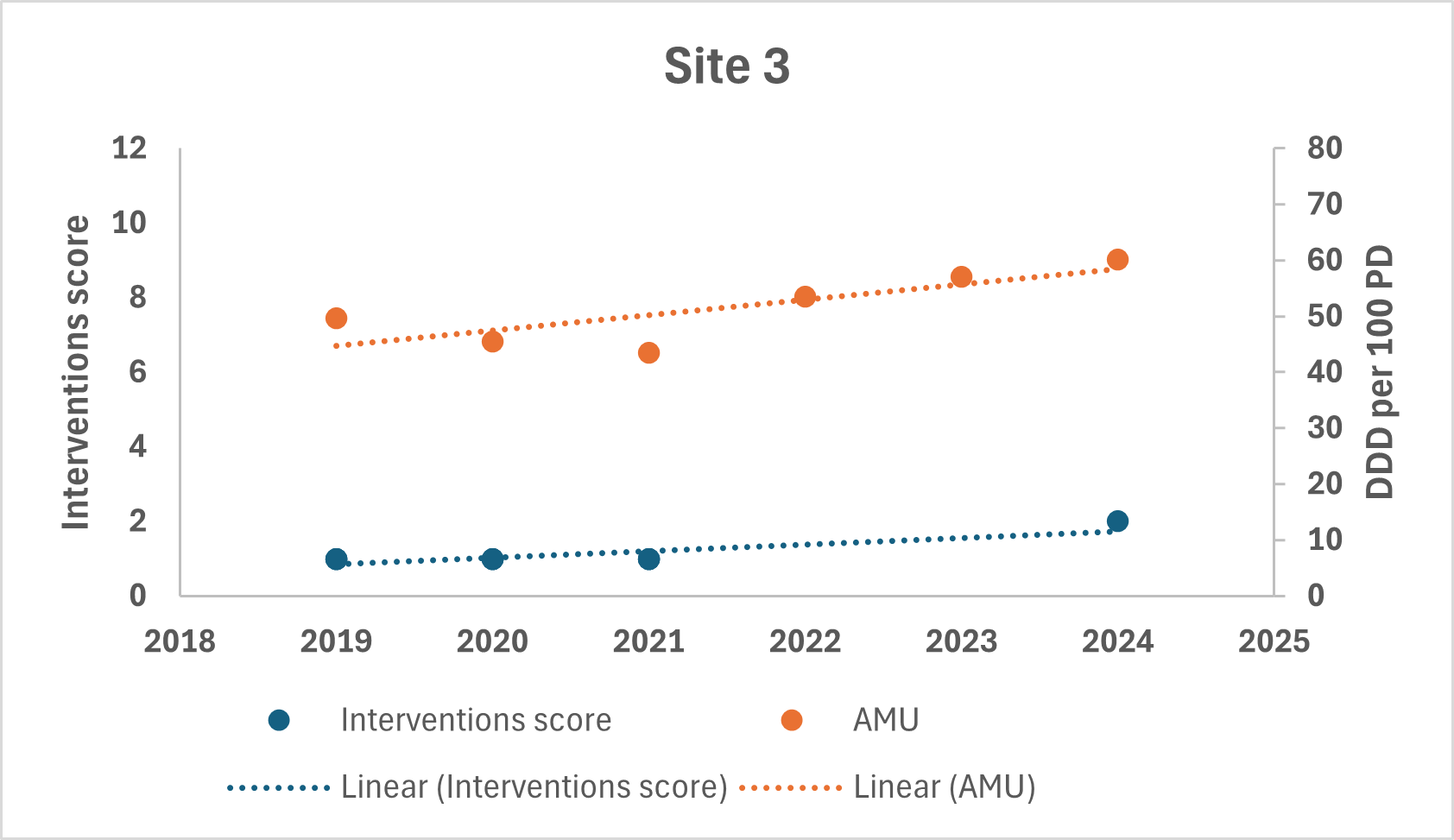

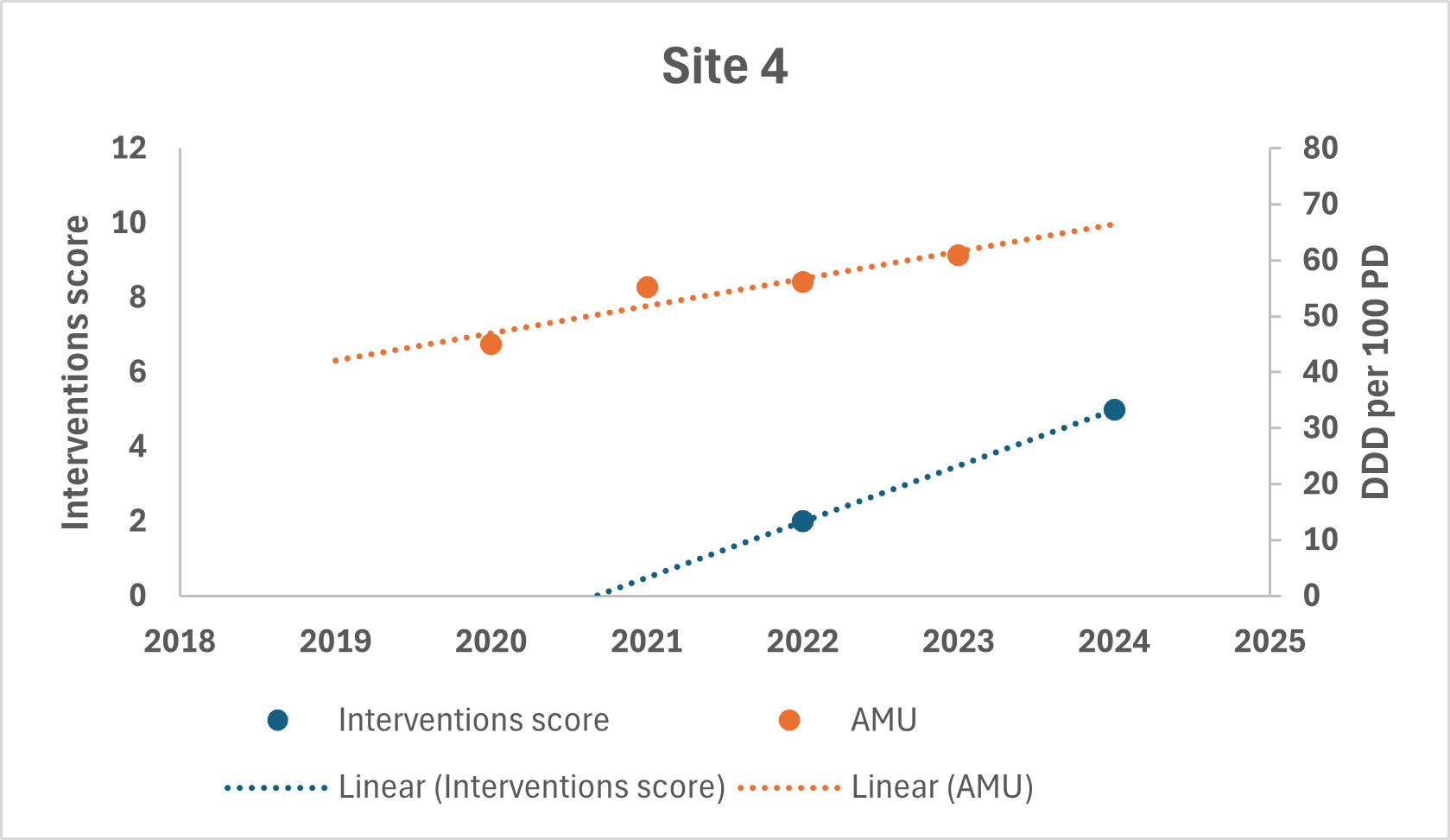


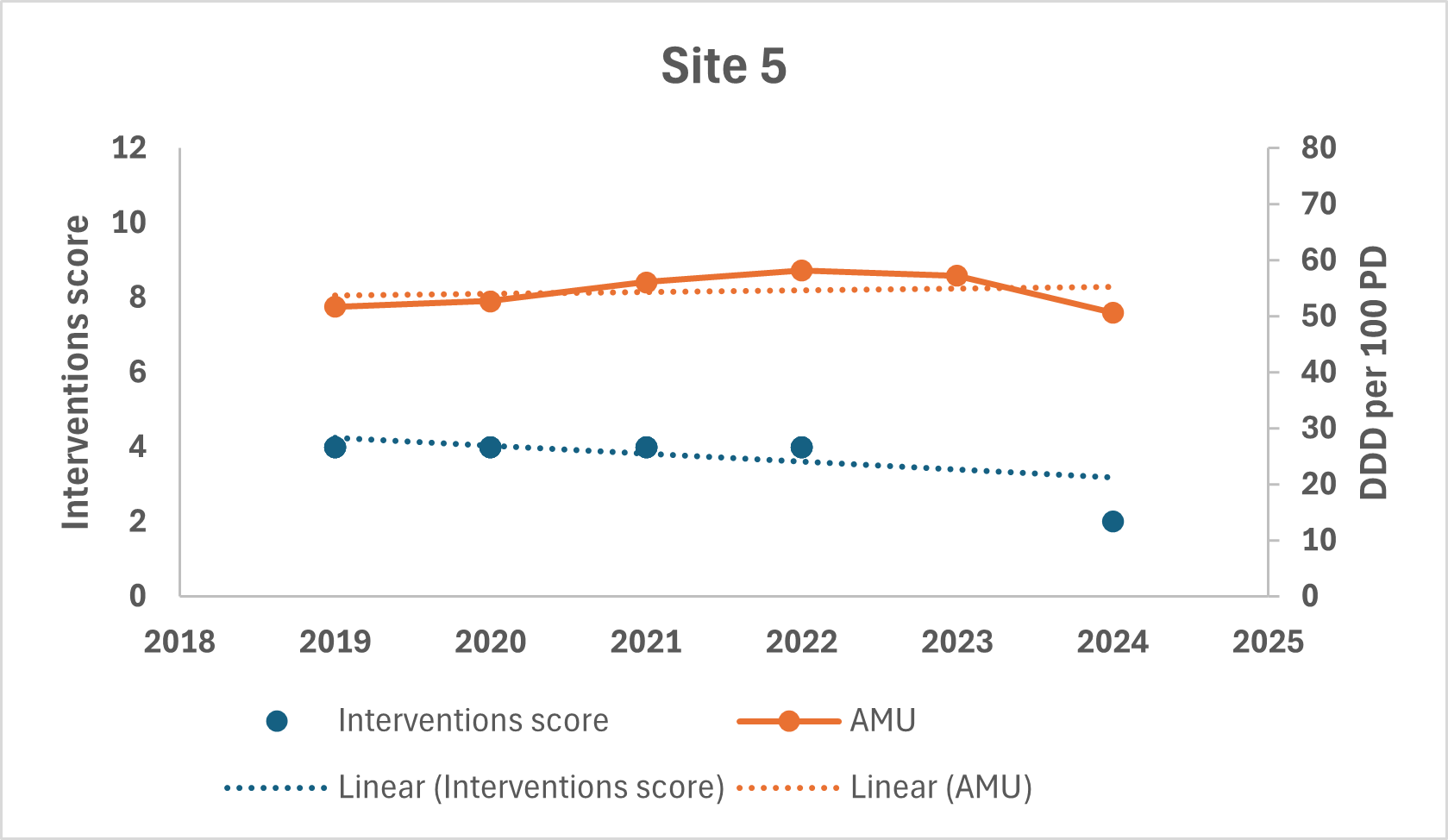

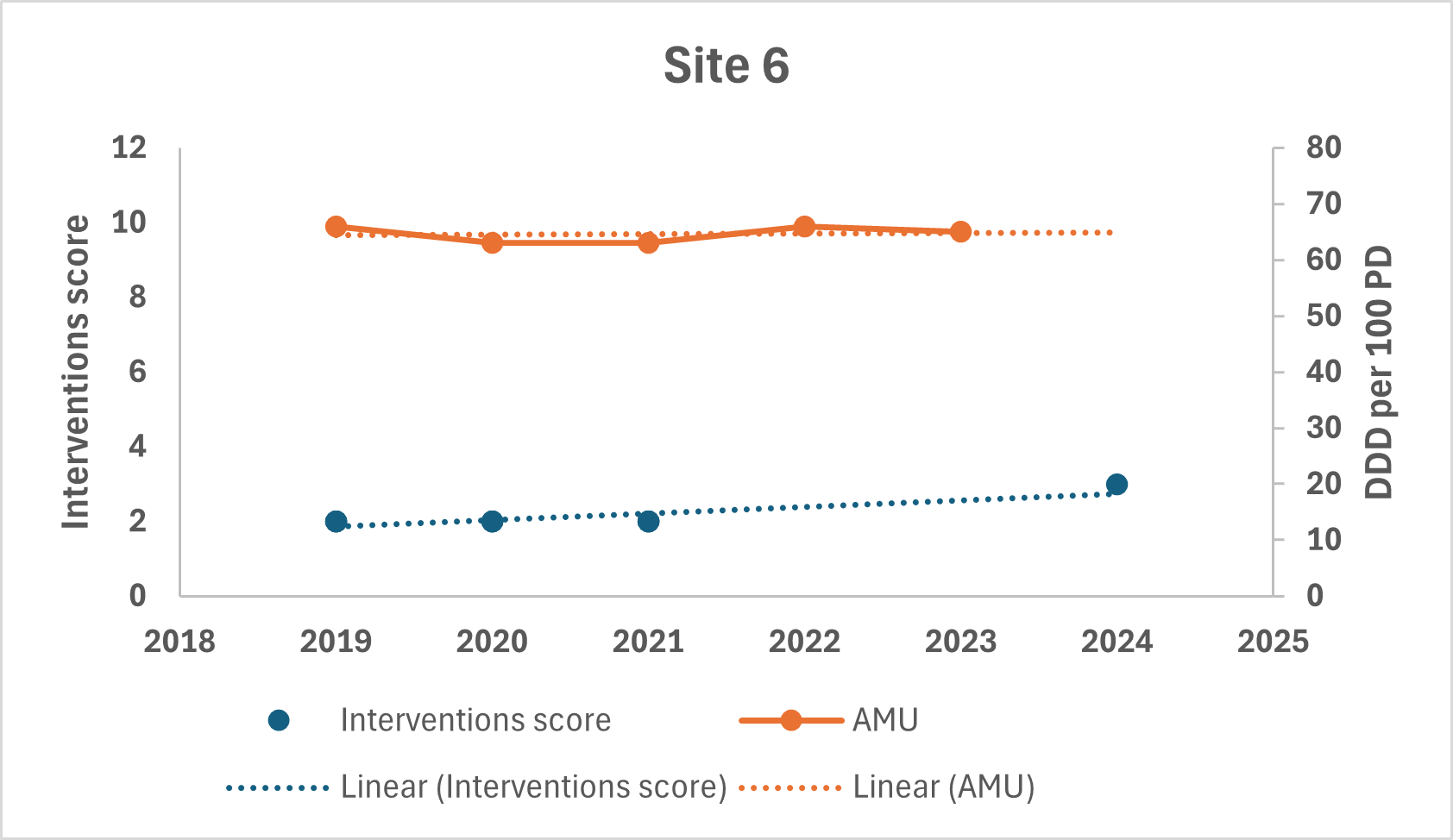


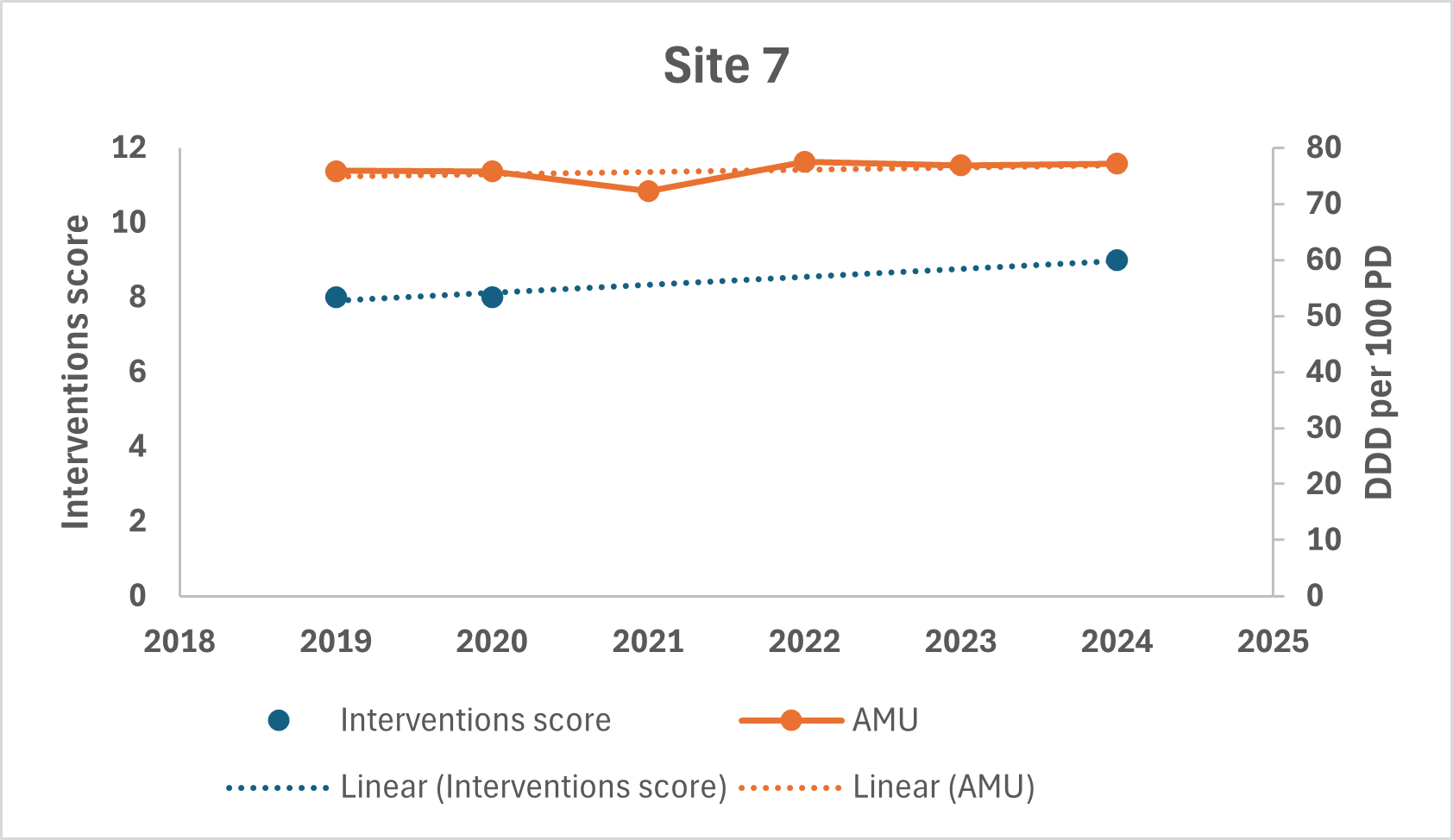

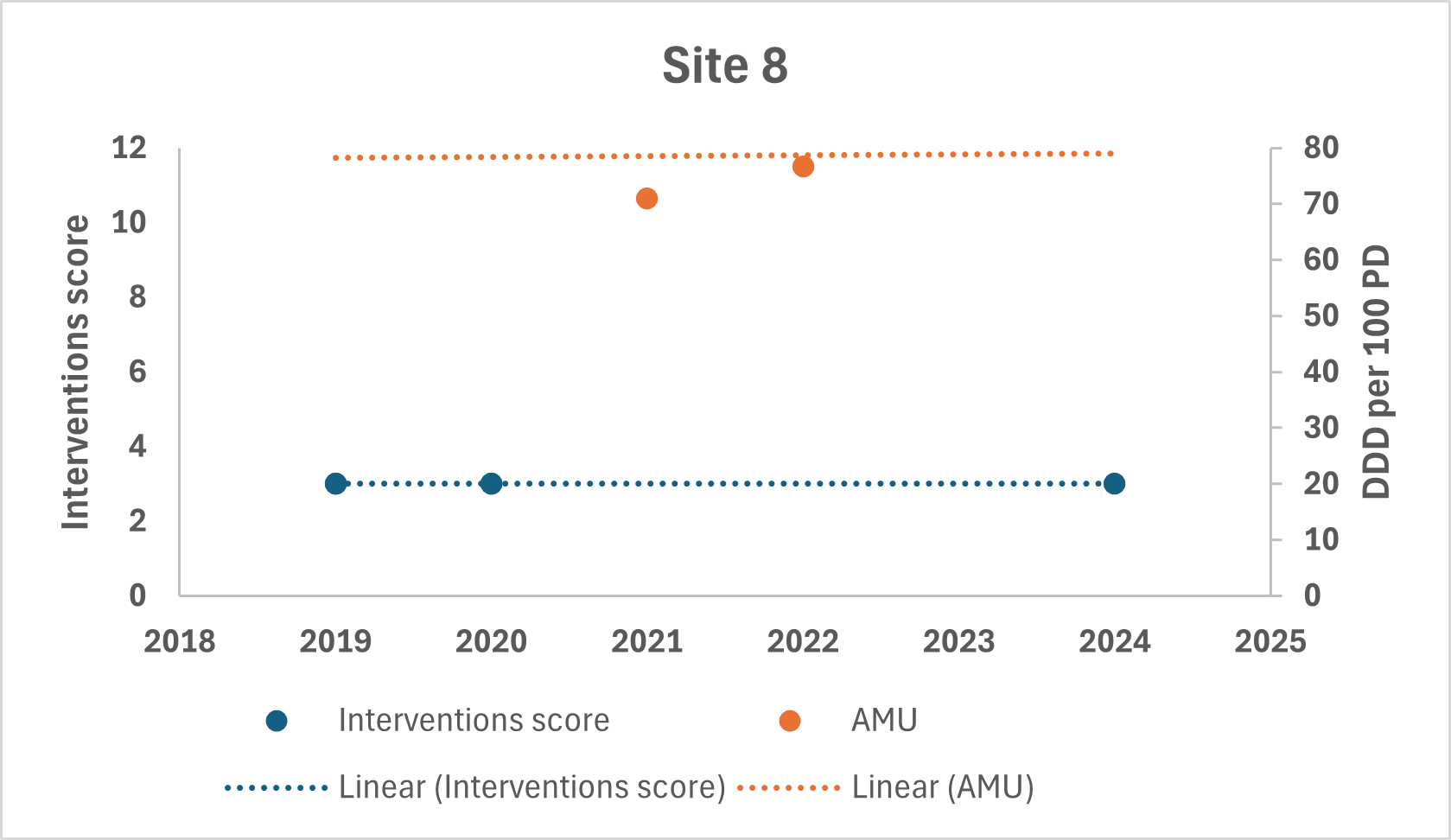


### Figure 4S: Association between incidence of *C difficile* infections and scores


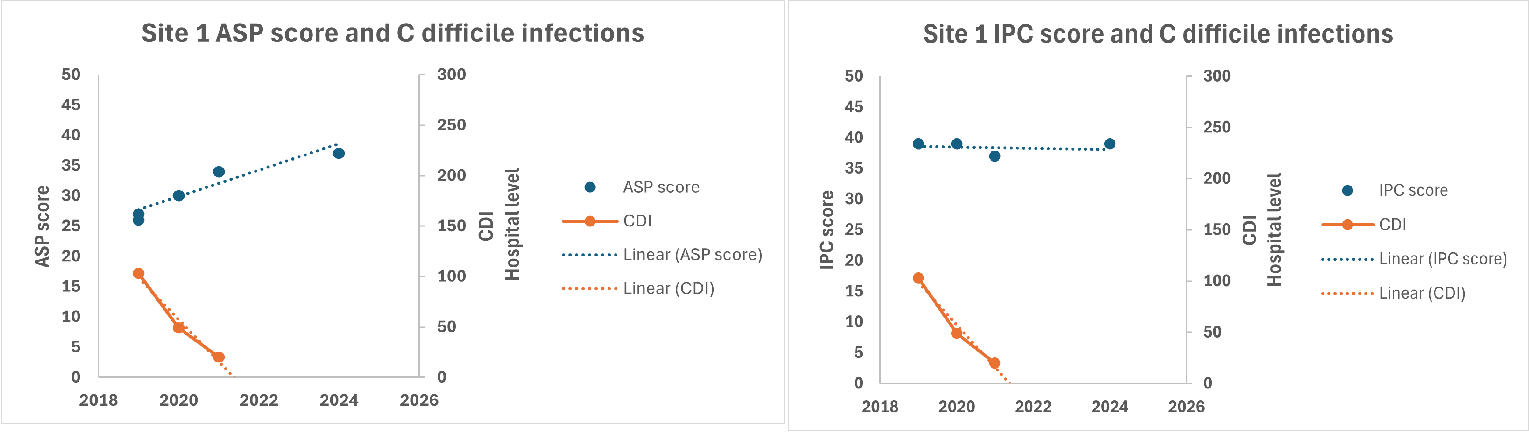


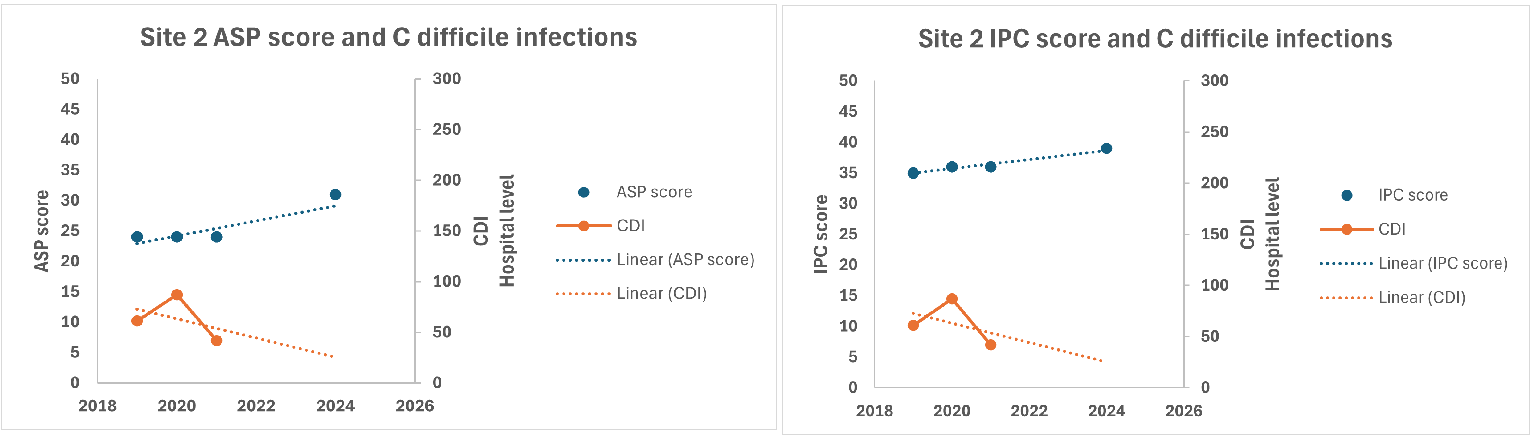


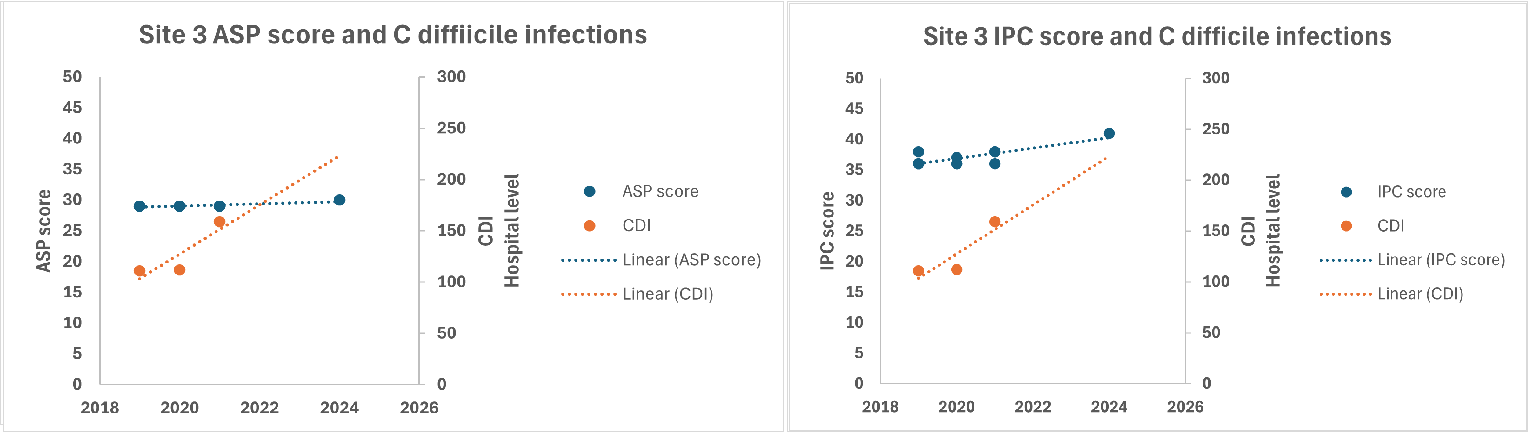


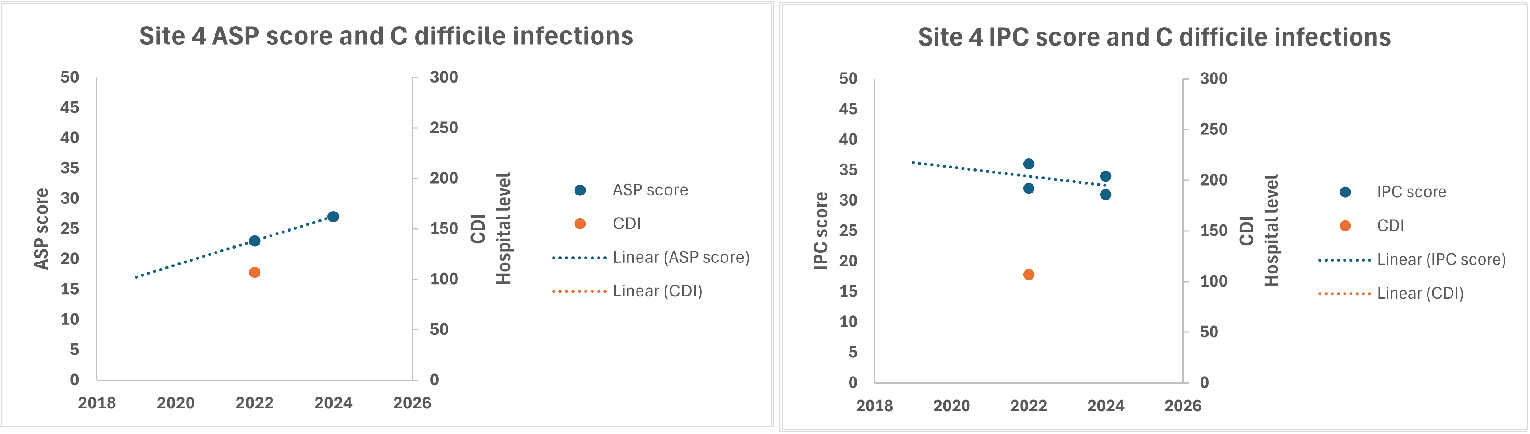


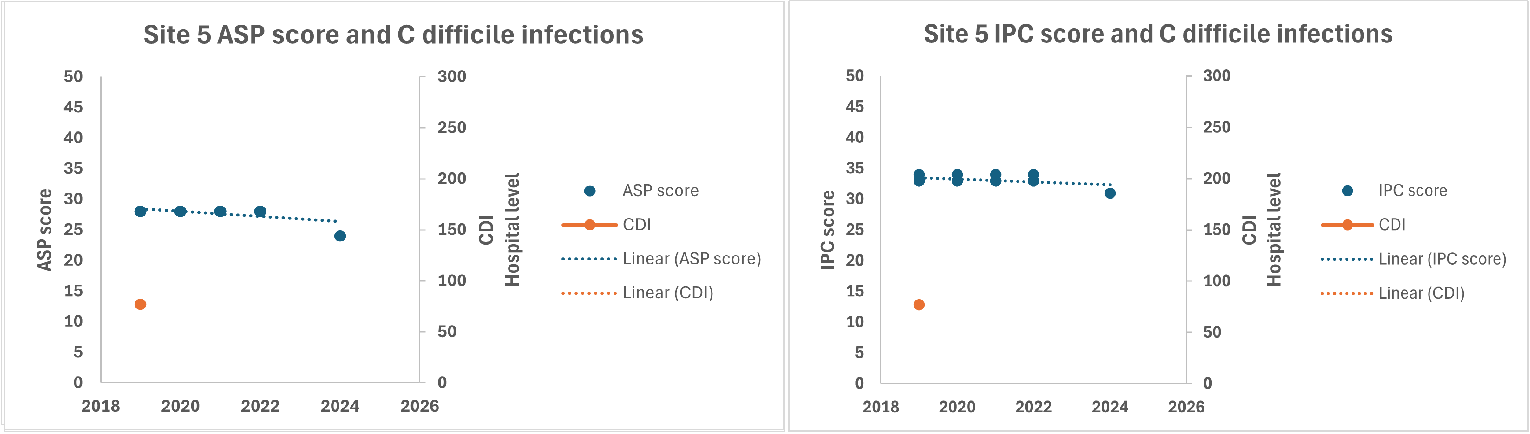


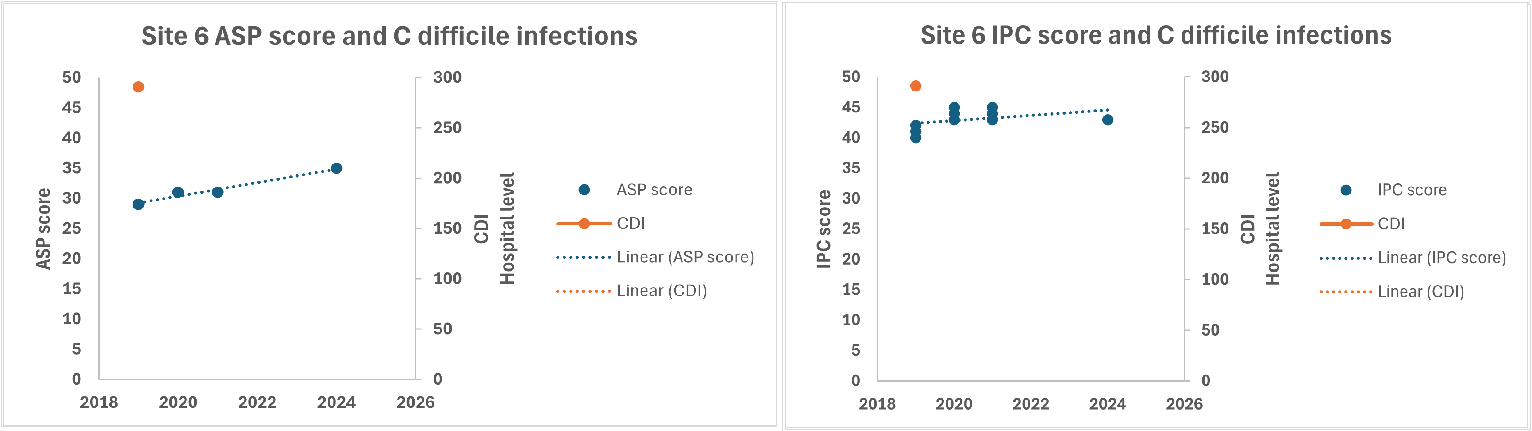


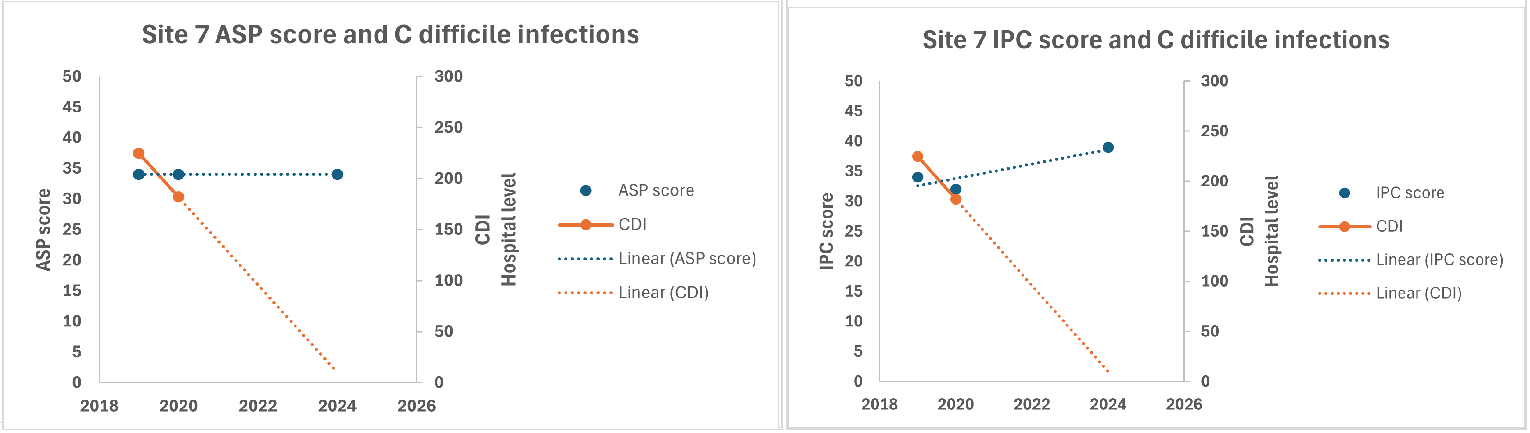


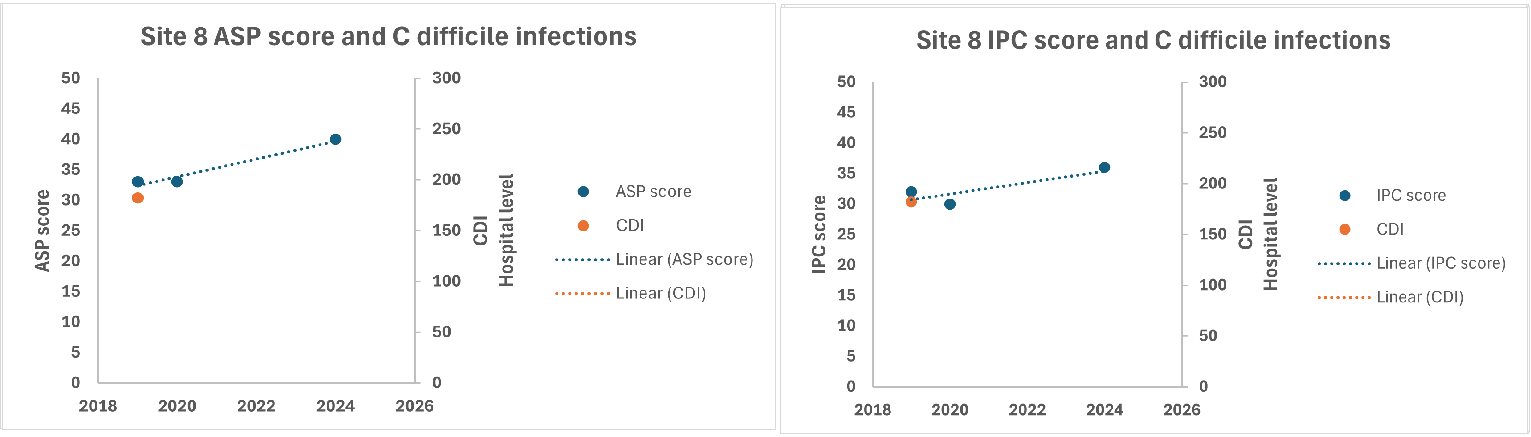


*C. difficile* infections are reported as absolute number of cases per year (hospital-wide). Sites 4 and 6 only reported data for one year.

### Figure 5S: Infection and Prevention Control (IPC) scores and hand disinfectant consumption


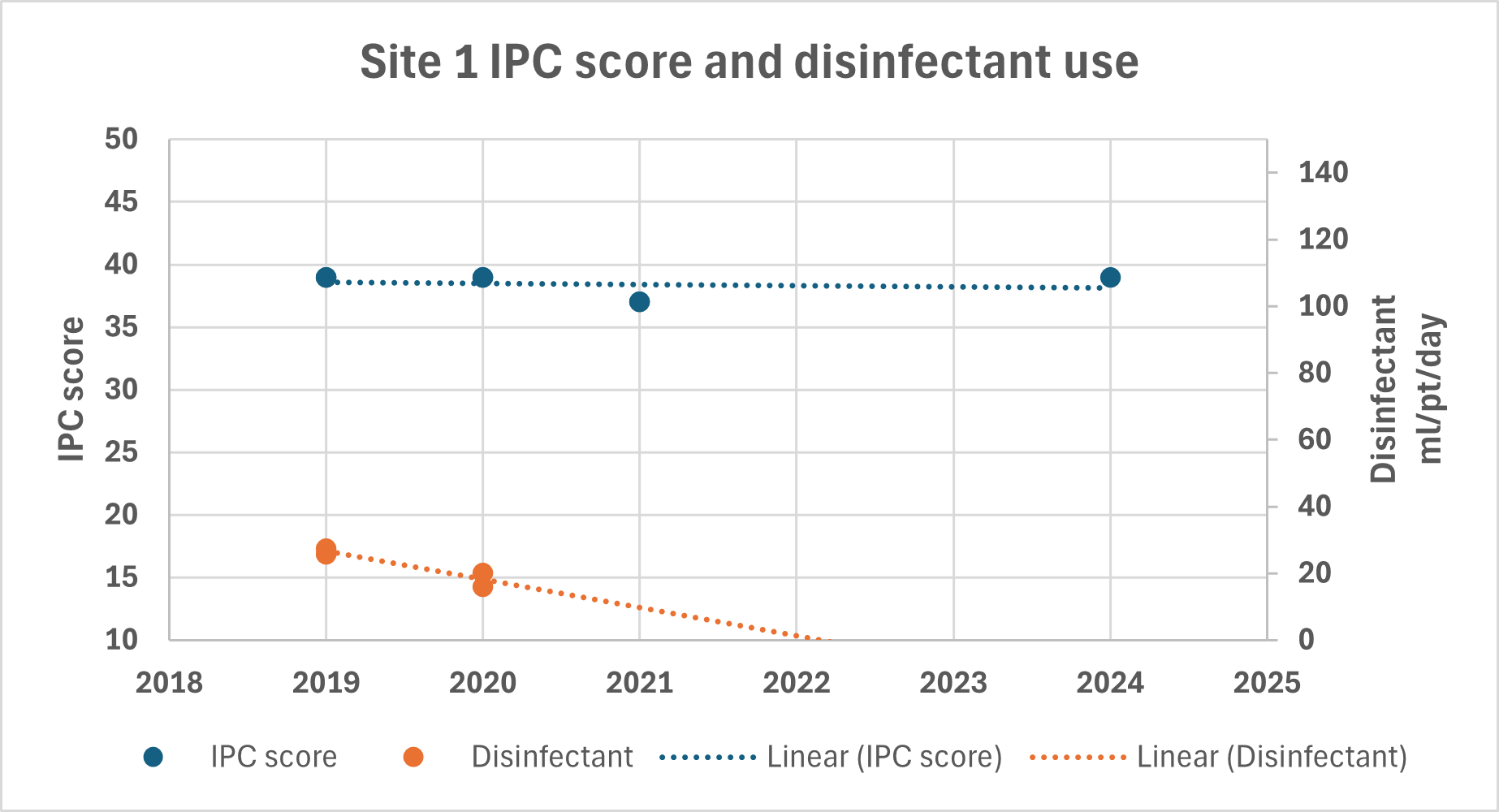

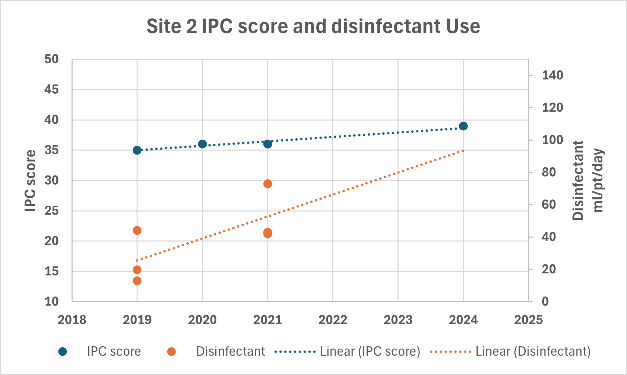


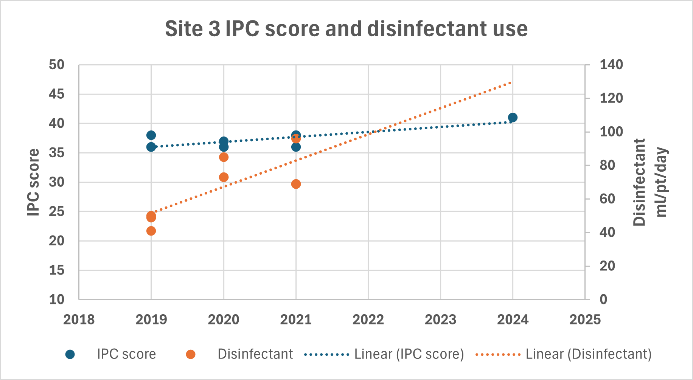

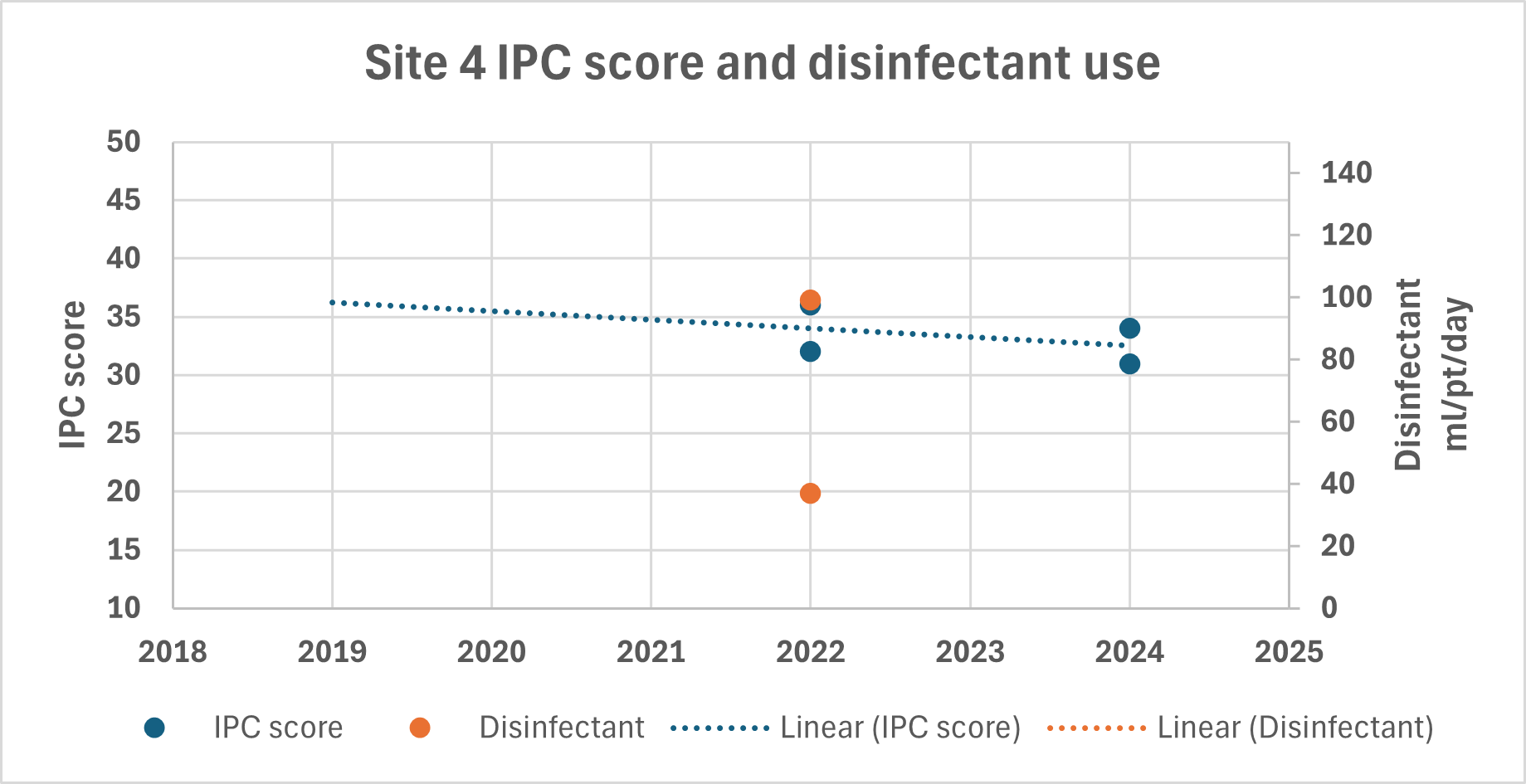


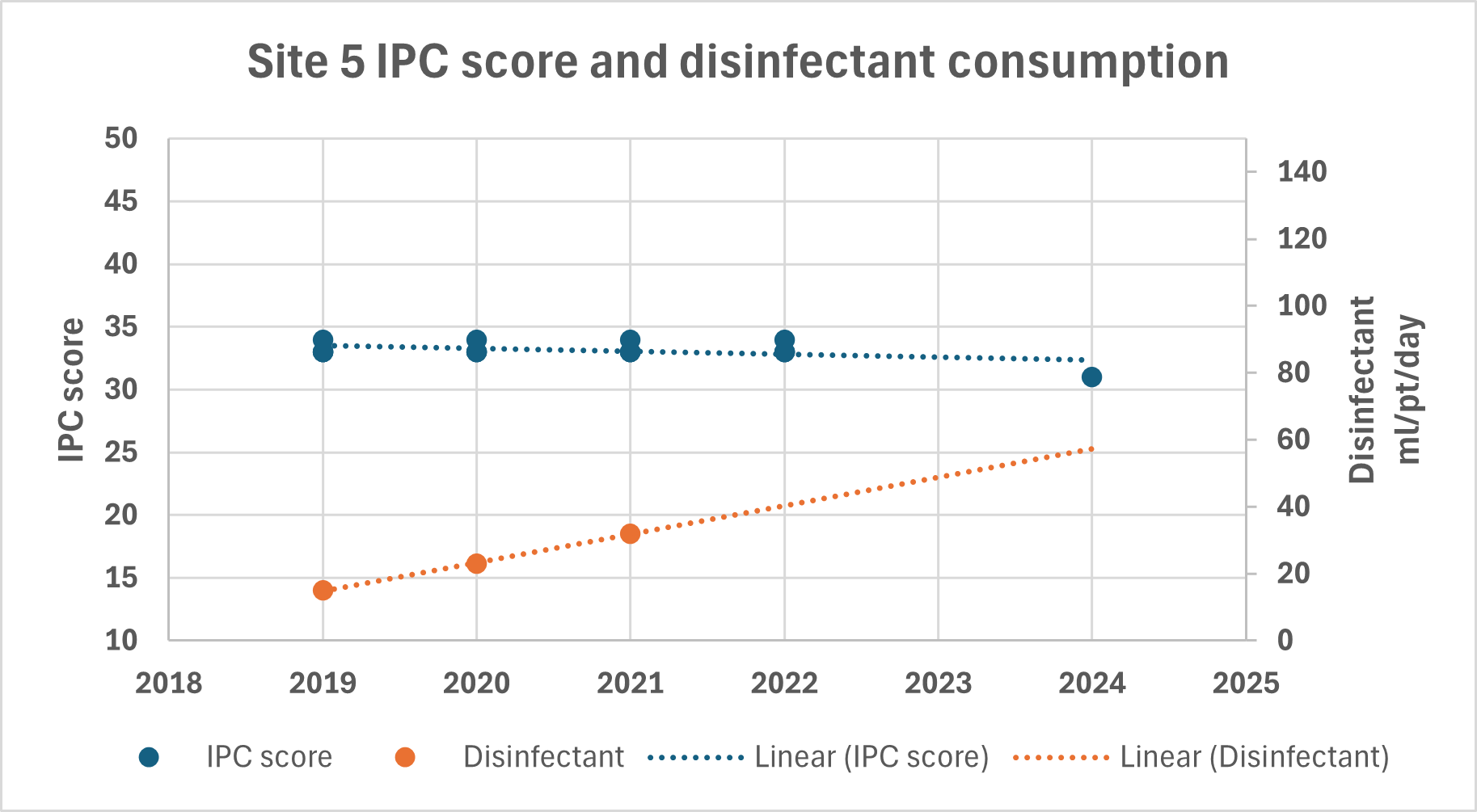

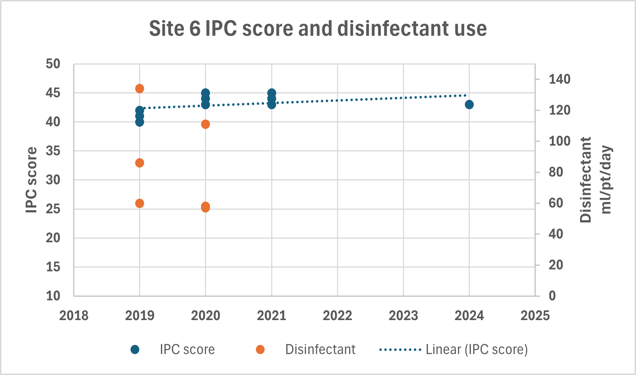


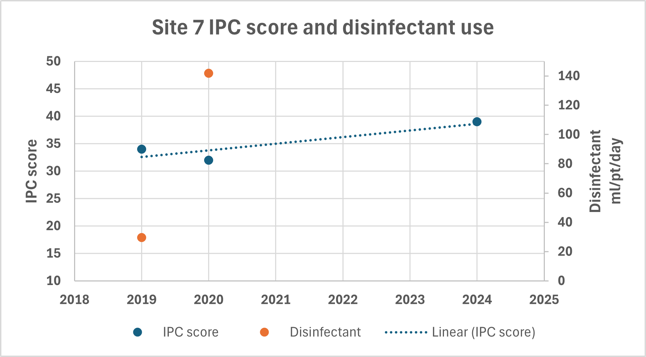

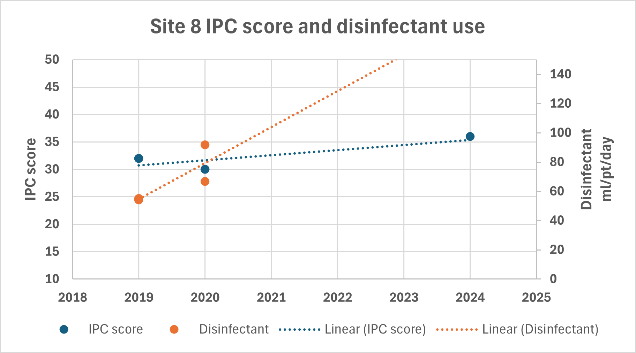


### Figure 6S: Hand Hygiene scores versus total Infection Prevention and Control scores


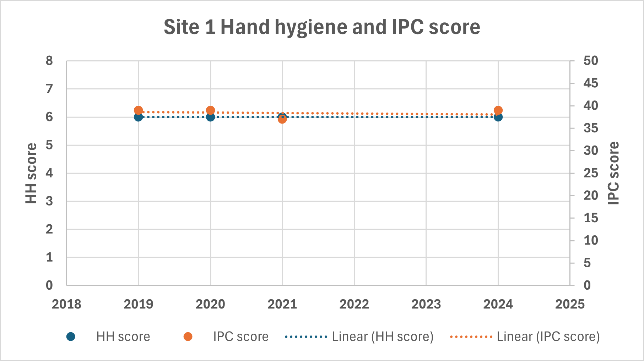

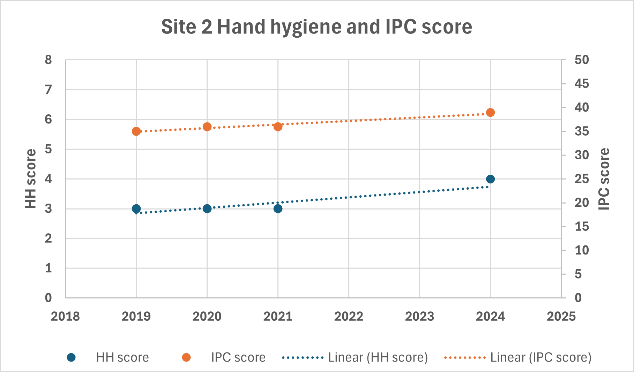


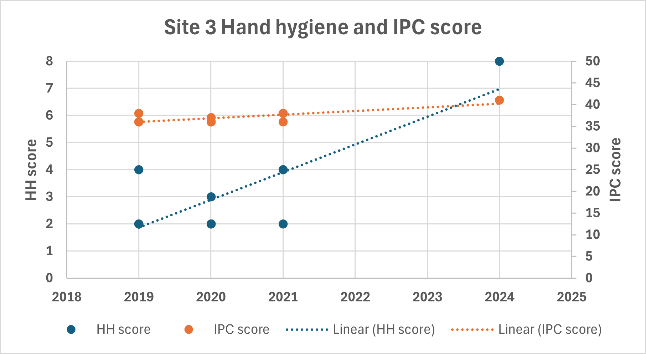

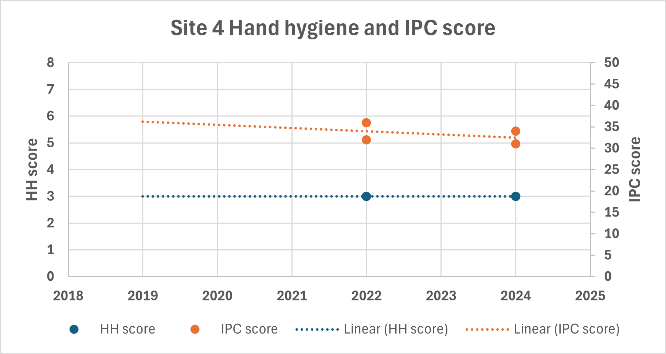


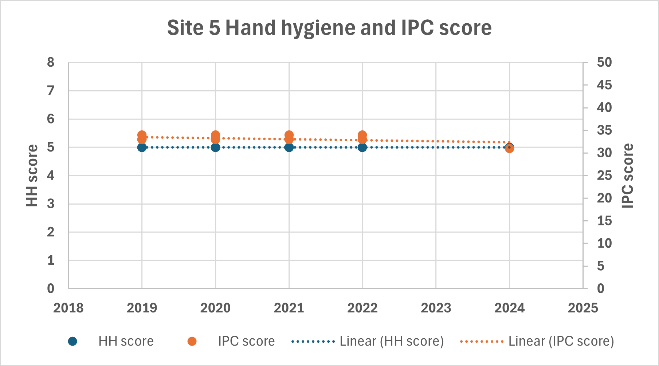

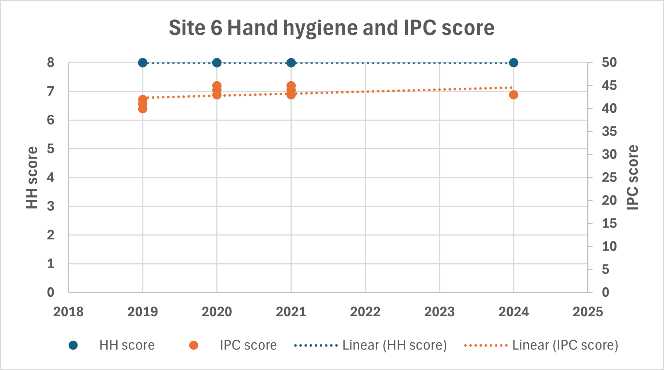


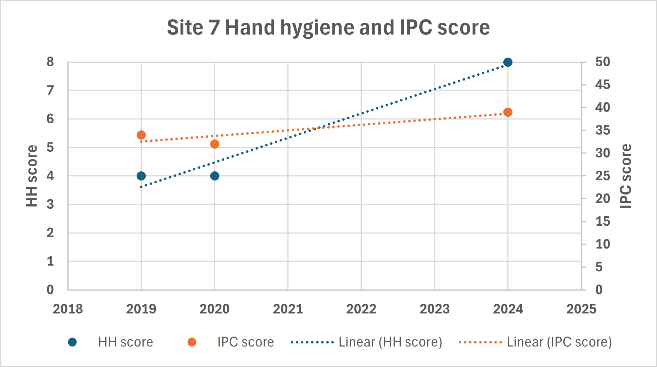

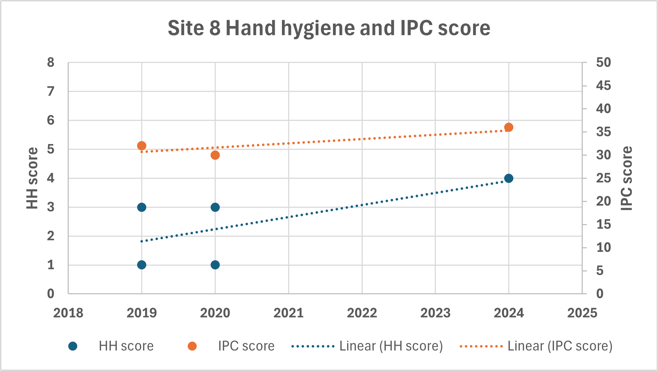

Supplement: dlag013_Supplementary_Data [file dlag013_supplementary_data.docx]
